# Pharmacotherapy of Traumatic Childhood Aphasia: Beneficial Effects of Donepezil Alone and Combined With Intensive Naming Therapy

**DOI:** 10.3389/fphar.2020.01144

**Published:** 2020-07-31

**Authors:** Guadalupe Dávila, María Pilar Moyano, Lisa Edelkraut, Lorena Moreno-Campos, Marcelo L. Berthier, María José Torres-Prioris, Diana López-Barroso

**Affiliations:** ^1^Cognitive Neurology and Aphasia Unit, Centro de Investigaciones Médico-Sanitarias, University of Malaga, Malaga, Spain; ^2^Instituto de Investigación Biomédica de Málaga - IBIMA, Málaga, Spain; ^3^Department of Psychobiology and Methodology of Behavioural Sciences, Faculty of Psychology and Speech Therapy, University of Malaga, Malaga, Spain; ^4^Language Neuroscience Research Laboratory, Faculty of Psychology and Speech Therapy, University of Malaga, Malaga, Spain

**Keywords:** language, childhood aphasia, anomia, traumatic brain injury, donepezil, pharmacological treatment, intensive naming therapy

## Abstract

At present, language therapy is the only available treatment for childhood aphasia (CA). Studying new interventions to augment and hasten the benefits provided by language therapy in children is strongly needed. CA frequently emerges as a consequence of traumatic brain injury and, as in the case of adults, it may be associated with dysfunctional activity of neurotransmitter systems. The use of cognitive-enhancing drugs, alone or combined with aphasia therapy, promotes improvement of language deficits in aphasic adults. In this study we report the case of a 9-year-old right-handed girl, subject P, who had chronic anomic aphasia associated with traumatic lesions in the left temporal-parietal cortex. We performed a single-subject, open-label study encompassing administration of the cholinergic agent donepezil (DP) alone during 12 weeks, followed by a combination of DP and intensive naming therapy (INT) for 2 weeks and thereafter by a continued treatment of DP alone during 12 weeks, a 4-week washout period, and another 2 weeks of INT. Four comprehensive language and neuropsychological evaluations were performed at different timepoints along the study, and multiple naming evaluations were performed after each INT in order to assess performance in treated and untreated words. Structural magnetic resonance imaging (MRI) was performed at baseline. MRI revealed two focal lesions in the left hemisphere, one large involving the posterior inferior and middle temporal gyri and another comprising the angular gyrus. Overall, baseline evaluation disclosed marked impairment in naming with mild-to-moderate compromise of spontaneous speech, repetition, and auditory comprehension. Executive and attention functions were also affected, but memory, visuoconstructive, and visuoperceptive functions were preserved. Treatment with DP alone significantly improved spontaneous speech, auditory comprehension, repetition, and picture naming, in addition to processing speed, selective, and sustained attention. Combined DP-INT further improved naming. After washout of both interventions, most of these beneficial changes remained. Importantly, DP produced no side effects and subject P attained the necessary level of language competence to return to regular schooling. In conclusion, the use of DP alone and in combination with INT improved language function and related cognitive posttraumatic deficits in a child with acquired aphasia. Further studies in larger samples are warranted.

## Introduction

Childhood aphasia (CA) is defined as a language impairment that affects previously acquired linguistic abilities, which cannot be explained by other cognitive or physical disorders (Aram, 1998). Since the diagnosis of CA requires a minimum development of linguistic skills prior to the brain injury, the age of 2 years is the established cut-off to differentiate CA from language developmental disorders (Woods and Teuber, 1978; Aram et al., 1985; Van Hout, 1997; Van Hout, 2003; Avila et al., 2010).

CA exhibits some singularities that distinguishes it from adult aphasia and raises the need for developing specific lines of research that take into account the characteristics of this population. Among these differences is the fact that brain damage during childhood may not only affect previously acquired language functions but also interfere with the ongoing brain maturation and language development. A further relevant differential feature is related to the etiology, thus while stroke is the leading cause of adult aphasia, the main cause of cognitive disability and aphasia in children and adolescents is traumatic brain injury (TBI) (Rothenberger, 1986; Jennett, 1996; Sergui-Gomez and MacKenzie, 2003; Babikian and Asarnow, 2009). Relevantly, one third of children that suffer a severe TBI, as measured by the Glasgow Coma Scale (Teasdale and Jennett, 1976), exhibit residual cognitive and language deficits (Anderson et al., 2001; Anderson et al., 2005; Anderson and Catroppa, 2006; Anderson et al., 2009) that may persist in the long term. Language disorders such as aphasia have a tremendous impact in the cognitive, social, and emotional development in children and adolescents, often resulting in reduced social integration, poor academic achievement, and behavioral problems (Beitchman et al., 2001; Johnson et al., 2010), as well as an increased risk of developing anxiety and social isolation during adulthood (Brownlie et al., 2016).

TBI usually results in focal and diffuse brain damage causing a wide range of linguistic deficits that may be contingent on several variables such as age at the time of injury, lesion size and location, severity of the injury, as well as premorbid language functioning (Sullivan and Riccio, 2010). Axonal injury derived from diffuse damage emerges as a result of the sudden acceleration and deceleration forces together with the simultaneous rotation of the freely moving brain mass (Levin, 2003; Vik et al., 2006). Importantly, diffuse axonal injury frequently affects white matter bundles connecting frontal and temporal cortical areas (Levin, 2003; Vik et al., 2006) that support linguistic and executive functions, including attentional capacity and processing speed. Accordingly, linguistic deficits in CA are frequently associated with weakened executive functions, such as deficits in lexical access as observed in naming tasks (Coelho, 2007; Slomine and Locascio, 2009). In fact, word finding difficulties (i.e. anomia) in spontaneous speech, naming and fluency tasks (Laine and Martin, 2006) are common deficits in the medium and long term after TBI and may persist even when other domains have been recovered (Van Hout et al., 1985; Narbona and Crespo-Eguilaz, 2012). However, despite its high frequency (Ewing-Cobbs and Barnes, 2002), reported cases of CA resulting from TBI are scarce, probably because in many cases linguistic deficits are hidden behind general cognitive impairments (e.g., attention).

At brain level, language and executive functions depend on the activity of distributed networks involving bilateral dorsal and ventral structures. Current models suggest that language functions are supported by two functionally and anatomically segregated processing streams: dorsal and ventral (Hickok and Poeppel, 2000). On the one hand, the dorsal stream is involved in verbal production and repetition (Saur et al., 2008) required, for instance, for phonological word learning (López-Barroso et al., 2013). This stream is supported by the arcuate fasciculus (AF) system connecting frontal, postero-temporal, and infero-parietal areas (Hickok and Poeppel, 2007). On the other hand, the ventral stream projects from the superior temporal gyrus to the middle and inferior temporal cortices to support semantic and comprehension processes (see Hickok and Poeppel, 2007). This ventral interaction occurs mainly through the inferior fronto-occipital fasciculus (IFOF), the inferior longitudinal fasciculus (ILF) and the uncinate fasciculus (UF) (Catani and Thiebaut de Schotten, 2008). Despite the above-mentioned functional division of labour, the language system is flexible enough to recruit additional areas during high-demanding language situations (Lopez-Barroso et al., 2011; Torres-Prioris et al., 2020) or during development, when some pathways are not fully mature (Brauer et al., 2011). For instance, studies of children with brain injury have shown that early damage to the AF may be succesfully compensated through recruitment of ipsilateral and contralateral brain areas and tracts, resulting in an average performance on multiple language tasks (Rauschecker et al., 2009; Asaridou et al., 2020), although some deficits may persist (Yeatman and Feldman, 2013). In this line, after early brain damage, functional and structural rightward lateralization of the dorsal pathway is associated with better language outcomes (Northam et al., 2018; François et al., 2019). Despite this evidence, spontaneous readjustment of the language system after brain lesion seems to be limited as evidenced by the frequent persistence of language deficits (Tavano et al., 2009; Turkstra et al., 2015; François et al., 2016). Therefore, research aimed at developing effective interventions to potentiate language recovery in CA is highly needed.

Despite the increasing efforts to advance in the development of effective therapeutic strategies for the cognitive and language after-effects of childhood TBI, studies targeting modern treatment approaches (cognitive/language therapy, pharmacotherapy, non-invasive brain stimulation) in CA are still scarce (for a review on rehabilitation programs for children with acquired brain injury not focused on language, see Laatsch et al., 2007; Slomine and Locasio, 2009). The fact that there are so few studies on this topic is probably because interventions for CA are frequently tailored to individual cases and carried out in instructional settings (Bowen, 2005; Duff and Stuck, 2015) with no sound methodological designs. The few existing intervention studies mainly focused on exploring the efficacy of behavioral strategies, as well as on identifying compensatory behaviors (Sullivan and Riccio, 2010; Turkstra et al., 2015). Overall, these interventions have proven beneficial effect for intensive training (6 to 8 weeks) of different language skills (lexical retrieval, verbal comprehension, fluency, communication pragmatic) and cognitive functions (attention, executive functions) commonly affected in TBI (Thomas-Stonell et al., 1994; Wiseman-Hakes et al., 1998; Chapman et al., 2005). Yet, the results from these studies are variable and, despite the growing number of published reports on cognitive and behavioral deficits after childhood TBI, rehabilitation recommendations are still insufficient.

The well-established strategy of using cognitive-enhancing drugs alone or in combination with speech-language therapy in adults with post-stroke aphasia (see Berthier and Pulvermuller, 2011; Berthier et al., 2011) has not been explored in CA. In adult post-stroke aphasia, several clinical trials have shown that a combined intervention with the cholinesterase inhibitor donepezil (DP) and speech-language therapy significantly improves language skills and communication (see Berthier et al., 2011; Zhang et al., 2018). The rationale for using cholinergic compounds to treat aphasia arises from the fact that brain lesions disrupt the cholinergic transmission from the basal forebrain and brainstem nuclei to the thalamus, basal ganglia, subcortical white matter, and cerebral cortex, including the left perisylvian language core (Simić et al., 1999; Mesulam, 2004; Mena-Segovia and Bolam, 2017; Markello et al., 2018). The resulting cholinergic depletion negatively influences learning, declarative memory, language, and attention by reducing experience-dependent neural plasticity to relevant stimuli during training (Kleim and Jones, 2008; Rokem and Silver, 2010; Gielow and Zaborsky, 2017). Although experimental TBI studies have shown that cholinergic neurotransmission is chronically depleted after TBI (Dixon et al., 1996; Dixon et al., 1997; Ciallella et al., 1998), the role of the cholinesterase inhibitor DP in adult TBI is controversial (Walker et al., 2004; Warden et al., 2006; Shaw et al., 2013) unless when used in combination with environmental enrichment therapies (*see* De la Tremblaye et al., 2019).

Accumulating evidence suggests that anticholinesterasic agents improve executive functions (Castellino et al., 2012; Castellino et al., 2014), sustained attention (Spiridigliozzi et al., 2007), learning, memory (Spiridigliozzi et al., 2007; Castellino et al., 2012), and language functions (Heller et al., 2004) in children. Importantly, the safety of these drugs in the pediatric population has been widely demonstrated (Biederman and Spencer, 2000; Hardan and Handen, 2002; Heller et al., 2004; Spiridigliozzi et al., 2007; Kishnani et al., 2010; Handen et al., 2011; Castellino et al., 2012; Sahu et al., 2013; Thornton et al., 2016). Over the last twenty years, the anticholinesterasic compound DP has been variously used to counteract impaired cognitive functions resulting from oncologic treatment of pediatric brain tumors (Castellino et al., 2012; Castellino et al., 2014; Lassaletta et al., 2015); to improve the core symptoms of attention-deficit/hyperactivity disorder (Biederman and Spencer, 2000; Popper, 2000; Wilens et al., 2000; Banaschewski et al., 2004; Pityaratstian, 2005) and of inattention-hyperactivity and communication abnormalities in autism spectrum disorders (Doyle et al., 2006; Hazell, 2007; Tamasaki et al., 2016). However, up to now there are no intervention studies aimed to explore the efficacy of cognitive-enhancing drugs, such as DP, to ameliorate language and cognitive deficits in CA.

The main aim of the present study was to evaluate the effects of pharmacotherapy with DP alone and combined with intensive naming therapy (INT) on CA recovery. Our secondary objective was to examine the effects of both interventions on naming, reading, and other linguistic and cognitive functions (executive functions, attention, memory), which were expected to change due to the interventions. Finally, the impact of TBI on the brain structure was explored at baseline to describe the possible brain-behavior relationship in light of the current knowledge. To do that, we studied the case of a 9-year old girl (subject P) with posttraumatic chronic anomic aphasia who was evaluated and treated following a single-subject, open-label design with DP alone and in combination with INT. DP was selected because it has repeatedly been shown to be effective in reducing aphasia severity, but also in boosting performance on lexical retrieval tasks (picture naming) in post-stroke aphasic adults (Berthier et al., 2003; Berthier et al., 2006). In addition, it is well known that the effect of cholinergic stimulation is more powerful when it is combined with behavioral training to promote experience-dependent plasticity (Berthier et al., 2014; Berthier et al., 2017). INT was selected because previous works have demonstrated that short-term intensive language therapies are more effective than distributed therapies (Pulvermuller et al., 2001; Kurland et al., 2010; Berthier et al., 2014). Considering previous evidence, it was expected that DP alone would induce significant improvements in attentional and executive functions and, as a result, language functions would be enhanced. Further gains were expected in language, attentional, and executive functions with the synergistic action of combined treatment with DP and INT. Since we also envisioned that gains in naming would be maintained after INT, several post-therapy evaluations were performed. To our knowledge, this is the first study evaluating the effects of DP and INT on language recovery in CA after TBI.

## Materials and Methods

### Case Description

**Figure 1:**
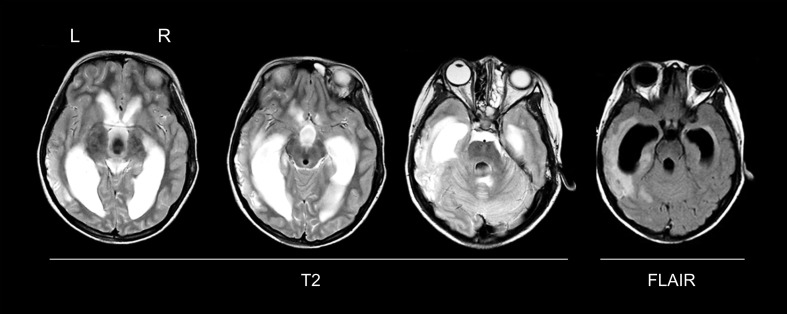
Structural magnetic resonance imaging (MRI) in the acute stage. A MRI (T2-weighted, and FLAIR sequences) was performed 4 days after traumatic brain injury, showing a marked communicating hydrocephalus with transependymal edema and a large area of contusion in the left lateral temporal lobe extending into the parahippocampal gyrus. Axial slices in native space are shown. L, left; R, right.

Subject P was a 9-year-old right-handed girl [+ 100 on the Edinburgh Handedness Inventory (Oldfield, 1971)] who suffered a severe closed TBI after being hit by a car on a pedestrian crossing. At the time she was admitted to the emergency room of a local Pediatric University Hospital, she was in profound state of coma, with bilateral otorrhagia, and a right hemiparesis. An emergency computerized tomography scan of the brain revealed diffuse bilateral brain edema, peribrainstem subarachnoid hemorrhage, and a focus of contusion in the left temporal-parietal region. A structural brain magnetic resonance imaging (MRI) 4 days later (acute stage) revealed marked communicating hydrocephalus and left temporo-parietal and parahippocampal non-hemorrhagic contusions (**Figure 1**). The hydrocephalus was uneventfully resolved with a ventriculo-peritoneal shunt. In the following days, subject P presented a gradual recovery of consciousness that uncovered a pronounced language impairment. Bedside language testing revealed that she was mute with null comprehension but had severe automatic echolalia, a profile compatible with mixed transcortical aphasia (Berthier, 1999). The aphasia and the right hemiparesis improved and subject P was referred to our Unit for evaluation of residual language deficits 6 months after the TBI. Her parents contacted the research team after reading about our work on combined treatments by using cognitive-enhancing drugs and aphasia therapy to treat acquired language disorders. Subject P was of Chinese origin, being adopted at the age of 4 by a Spanish couple living in Malaga, Spain. Her medical records from China indicated that she had no medical problems and showed typical motor, cognitive, and language developmental milestones. At the time of adoption, subject P only spoke Chinese, but she rapidly learned Spanish and started using it both at school and at home. At the time she suffered the TBI, she had normal language development and schooling records. She attended the third grade of elementary school, which was the academic course corresponding to her age.

### Study Design

**Figure 2:**
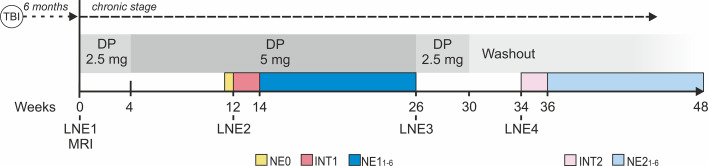
Study design. A single-subject, open-label longitudinal design with drug and language interventions was used. DP, donepezil; TBI, traumatic brain injury; INT, intensive naming therapy; LNE, language and neuropsychological evaluation (1-4); NE0, baseline naming evaluation in which naming performance for the full set of treated and untreated words used in INT1 and INT2 was evaluated; NE, naming evaluations (1-6) performed after each INT; MRI, magnetic resonance imaging.

A single-subject, open-label design was used. **Figure 2** depicts the study design. At the beginning of the trial (week 0), DP was started at a very low dose (2.5 mg/day) and titrated up to 5 mg/day one month after initiating the treatment (week 4). This DP dose was maintained and administered alone for 8 weeks (weeks 4 to 12) and then it was combined with INT (INT1) for 2 weeks (weeks 12 to 14). Thereafter, subject P continued treatment with DP alone (5 mg/day) for 12 weeks (weeks 14 to 26) and thereupon it was gradually tapered off over 4 weeks (weeks 26 to 30). This intervention phase was followed by a washout period of 4 weeks (weeks 30 to 34) and then by 2 weeks of INT alone (INT2) (weeks 34 to 36)^1^. Language and neuropsychological evaluations (LNE) were performed at different timepoints (LNE1, LNE2, LNE3, LNE4; as illustrated in **Figure 2**) in order to track the language and cognitive impact of the different interventions. In addition, to evaluate the duration of the potential gains achieved with each INT, six post-therapy naming evaluations (NE) were performed after each INT phase in which naming performance for treated and untreated control words was assessed. A baseline NE (NE0), including all treated and untreated words, was performed before INT1. Evaluations and language therapies were performed by the first author (GD), a neuropsychologist with experience in aphasia testing and treatment.

### Drug Treatment

The cholinergic agent DP was used according to the statement of ethical principles for medical research involving human subjects of the Declaration of Helsinki (section 37: Unproven Interventions in Clinical Practice). The protocol of this study was approved by the local Ethical Research Committee (Provincial of Malaga, Spain). DP has been used in several developmental and acquired cognitive and behavioral disorders involving children and adolescents (Biederman and Spencer, 2000; Popper, 2000; Wilens et al., 2000; Hardan and Handen, 2002; Spencer and Biederman, 2002; Heller et al., 2004; Pityaratstian, 2005; Doyle et al., 2006; Hazell, 2007; Spiridigliozzi et al., 2007; Cubo et al., 2008; Kishnani et al., 2010; Buckley et al., 2011; Handen et al., 2011; Srivastava et al., 2011; Castellino et al., 2012; Sahu et al., 2013; Castellino et al., 2014; Lassaletta et al., 2015; Tamasaki et al., 2016; Thornton et al., 2016). Treatment with DP in this population has proven to be safe (Biederman and Spencer, 2000; Hardan and Handen, 2002; Heller et al., 2004; Spiridigliozzi et al., 2007; Kishnani et al., 2010; Handen et al., 2011; Castellino et al., 2012; Sahu et al., 2013; Thornton et al., 2016), demonstrating good efficacy profile (Biederman and Spencer, 2000; Popper, 2000; Wilens et al., 2000; Hardan and Handen, 2002; Spencer and Biederman, 2002; Heller et al., 2004; Pityaratstian, 2005; Doyle et al., 2006; Hazell, 2007; Spiridigliozzi et al., 2007; Cubo et al., 2008; Buckley et al., 2011; Srivastava et al., 2011; Castellino et al., 2012; Castellino et al., 2014; Lassaletta et al., 2015). Therefore, we considered that the prescription of this agent for an unapproved use was appropriate for this particular case of CA. The dose of DP was chosen based on the prescription used in previous studies of DP in pediatric population (Kishnani et al., 2010; Srivastava et al., 2011; Sahu et al., 2013), on the child’s weight (Hardan and Handen, 2002; Kishnani et al., 2004; Castellino et al., 2012), and on the proven tolerability of this agent (Heller et al., 2004; Spiridigliozzi et al., 2007). Subject P’s parents were provided with the package leaflet of DP, and they were also fully informed about the pharmacological characteristics, the potential benefits, and adverse events of the drug. Written informed consent was obtained from subject P and her parents. During both the titration phase and the drug treatment, they were contacted regularly to detect potential adverse events and to track the adherence to the drug treatment.

### Intensive Naming Therapy (INT)

INT based on hierarchical cueing was administered 1.5 h per day, 7 days per week, first during 2 weeks combined with DP (INT1, **Figure 2**) and, after, during 2 more weeks administered alone in the DP washout phase (INT2, **Figure 2**), resulting in a total duration of 4 weeks (~ 42 h). Stimuli in each INT session were black and white pictures representing Spanish nouns presented on a computer screen. Naming therapy based on cueing hierarchy has been shown to be effective in the treatment of naming deficits (Fridriksson et al., 2005; Green-Heredia et al., 2009; Best et al., 2013; Suárez-González et al., 2015). After each picture was presented, subject P was required to name the depicted stimulus. If she could not name the target picture in 20 s, a written phonological cue (i.e., the first syllable of the stimulus name) was then presented beneath the picture, and the word stem was read aloud by the therapist. In those circumstances in which subject P was still unable to name the target word, the full written name was presented underneath the picture and was read aloud by the therapist. After hearing it, subject P was asked to repeat the word aloud.

A set of 153 pictures consisting of white line drawings of living beings and non-living things was selected, all of them represented nouns. Half of these stimuli were trained in the two INTs (INT1 and INT2), and the other half were used as control items. The selection of these pictures was based on two criteria: (i) pictures that subject P consistently failed to name in the naming tests included in LNEs prior to INT1 (LNE1 and LNE2); and (ii) pictures selected from her natural science textbook, subject in which her parents reported marked naming difficulties. Specifically, 117 items were selected from the following naming tests: the object naming subtest of the Western Aphasia Battery-Revised ([WAB-R], Kertesz, 2007), the Snodgrass and Vanderwart Object Pictorial Set ([SVPS], Snodgrass and Vanderwart, 1980), the Boston Naming Test ([BNT], Kaplan et al., 1983), the Nombela 2.0 Semantic Battery ([NSB], Moreno-Martínez and Rodríguez-Rojo, 2015), and two naming subtests of the Psycholinguistic Assessments of Language Processing in Aphasia ([PALPA 53 and PALPA 54], Kay et al., 1992; Valle and Cuetos, 1995). The pictures from her natural science book (36 items) were selected by the therapist.

The 153 stimuli were divided into two sets, one containing 77 pictures and the other one 76 pictures, which were used as the to-be-trained and control items, respectively, for the INT1 and INT2. Specifically, 37/77 pictures were trained (hereinafter *treated words*) in the INT1 phase and 40/77 pictures corresponded to the treated words in the INT2 phase. The remaining pictures (36/76 and 40/76) were used as control items (hereinafter *untreated words*) in the six NEs performed after INT1 and INT2, respectively. The sets of treated and untreated words were matched by frequency (INT1: t(71) = -0.233, *p* = 0.816; INT2: t(78) = -1.58, *p* = 0.119), number of phonemes (INT1: t(71) = -0.394, *p* = 0.694; INT2: t(78) = -0.477, *p* = 0.635), syllables (INT1: t(71) = 0.089, *p* = 0.930; INT2: t(78) = -0.648, *p* = 0.519) and semantic category. In each INT session, the full set of treated words assigned to each INT was presented twice. To avoid associative learning between items, the presentation order of the words was randomized. For this, 10 lists were created containing all the items assigned to each INT but with different presentation order. Two lists were used in each daily session.

### Language and Neuropsychological Evaluations

In order to assess treatment-induced changes, a set of primary and secondary outcome measures comprising language, executive functions, attention and memory functions were selected. In addition, visuoconstructive and visuospatial functions were measured only at baseline. Note that the same outcome measures were used for both interventions (DP alone and combined treatment of DP and INT), in line with the expected changes.

#### Outcome Measures

The primary outcome measures consisted on different measures of the WAB-R (Kertesz, 2007). Specifically, these were the aphasia quotient (WAB-R AQ) and the WAB-R subtests scores: information content and fluency in spontaneous speech, comprehension, repetition, and naming. Despite contributing to the WAB-R AQ, the different WAB-R subtests were also included individually as primary outcome measures in that they are sensitive to detect treatment-induced changes and may show a differentiated evolution pattern (Berthier et al., 2003; Berthier et al., 2006).

The secondary outcome measures included a set of tests selected to assess relevant aspects of language and other cognitive functions, especially attentional and executive functions. As for the primary outcome measures, the functions targeted by these tests were expected to improve with the treatments. The selected language tests were: (a) the SVOPS, BNT, and NSB to evaluate naming; (b) the Peabody: Picture Vocabulary Test (PPVT-III), Dunn et al. (2006) to evaluate comprehension *via* word-picture matching; (c) the PALPA (Kay et al., 1992; Valle and Cuetos, 1995) to evaluate repetition, naming, comprehension, and reading; (d) the Token Test- short version ([TT-sv], De Renzi and Vignolo, 1962) to evaluate comprehension of syntax and spatial relationships; (e) and the Controlled Oral Word Association Task ([COWAT], Borkowski et al., 1967) to assess phonological verbal fluency (see **Table 2**). These tests as well as the ones included as primary outcome measures were administered in each LNE. The selected memory and executive functions tests were: Memory and Learning Test ([TOMAL], Reynolds and Bigler, 1996) (LNE1 and LNE2), Digit Span ([WISC-V] of the Wechsler Intelligence Scales for Children-V, Wechsler, 2014) (LNE1, LNE2, LNE3); attention: d2 Attention Test ([d2 Test], Brickenkamp and Cubero, 2002) (all LNEs); Neuropsychological Evaluation of Executive Functions in Children ([ENFEN], Portellano et al., 2009) (LNE1, LNE2, LNE3), Stroop (Stroop, 1935) (LNE1, LNE2, LNE3), and Five-Digit Test ([FDT]Sedó, 2004) (all LNEs).

Furthermore, although they were not expected to change due to treatment and, therefore, were not considered primary or secondary outcome measures, visuoconstructive and visuoperceptive functions were also assessed at baseline to estimate premorbid cognitive functioning. For this purpose, the two following tests were used: Rey–Osterrieth Complex Figure ([ROCF], Osterrieth, 1944), Benton Laboratory of Neuropsychology Tests ([BLNT], Benton et al., 1994) (see **Table 2**). As some of the employed tests in the LNEs are widely used in the Spanish population but may not be familiar to the English speaking countries, a brief description of these tests is provided in the **Supplementary Material**.

### Naming Evaluations (NE)

First, a baseline naming assessment (NE0) comprising the full set of 153 words was performed after treatment with DP alone and before INT1 (**Figure 2**). Then, in order to track the maintenance of gains in naming performance for treated words and the potential generalization to untreated ones, multiple NEs were performed after each INT. Specifically, after INT1 and INT2, six NEs were performed: 20 min after the end of each INT (NE1_1_ and NE2_1_), and at days 2 (NE1_2_ and NE2_2_), 7 (NE1_3_ and NE2_3_), 21 (NE1_4_ and NE2_4_), 49 (NE1_5_ and NE2_5_), and 84 (NE1_6_ and NE2_6_) (**Figure 2**). In each NE, treated and untreated words were evaluated. The presentation order of the words in each NE was randomized. No feedback was provided to subject P during the NEs.

### Control Group

Since there are no normative data for most of the language tests used in the evaluation of subject P, a control group of healthy children (classmates and relatives of subject P) was recruited in order to obtain reference scores for these tests. The group was composed of 7 children (4 boys and 3 girls) matched with subject P for age (8.9 ± 0.69 years; range: 8-10 years; Crawford’s t, two-tailed = 0.136; *p* = 0.896), general intelligence (verbal IQ: subject P = 95; control group = 112.43 ± 20.02 [Crawford’s t, two-tailed = -0.814; *p* = 0.446]; non-verbal IQ: subject P = 103; control group = 115.57 ± 15.43; [Crawford’s t, two-tailed = -0.762; *p* = 0.475]) and sociocultural background. Control children were administered the WAB-R and other tests (SVOPS, NSB, PALPA, and COWAT). Healthy adults tend to show a ceiling effect on the WAB-R AQ (AQ ≥ 93.8/100), and subjects with scores below this cut-off are considered to have aphasia. Regarding WAB-R use in children, it was reasoned that healthy children with ages between 8 and 10 years and high verbal intelligence quotient (IQ) (≥ 110) would have a good performance in the WAB-R.

The parents of the control children were informed about the aim of the study and written informed consent was obtained.

### Structural Neuroimaging

#### Image Acquisition

The MRI acquisition was performed at baseline (6 months after TBI) on a 3-T MRI scanner (Philips Gyroscan Intera, Best, The Netherlands) equipped with an eight-channel Philips SENSE head coil. Head movements were minimized using head pads and a forehead strap. Three-dimensional magnetization prepared rapid acquisition gradient echo (3D MPRAGE) was performed with the following parameters: acquisition matrix, 268/265; field of view, 224 mm; repetition time (TR), 9.2 ms; echo time (TE), 4.2 ms; flip angle, 8o; turbo field echo (TFE) factor, 200; reconstruction voxel size, 0.68 mm x 0.68 mm x 0.8 mm. Two hundred ten contiguous slices were acquired, with a 0 mm slice gap, the total acquisition time of the sequence was about 2 min and 50 s.

#### Lesion-Based Approach to Mapping Disconnection

Two different methods were used to gain knowledge about the direct and remote structural effects of the brain lesion. Tractotron and Disconnectome Maps, included in the BCB Toolkit (http://toolkit.bcblab.com/; Foulon et al., 2018).

In order to apply these methods, subject P’s lesion was manually delineated over the T_1_-weighted image in native space using MRIcron software (Rorden and Brett, 2000). Then, both the T_1_-weighted image and the binarized lesion mask were normalized to the MNI space using Statistical Parametric Mapping 12 (SPM 12, www.fil.ion.ucl.ac.uk/spm/). The normalized lesion was mapped onto tractography reconstructions of white matter pathways obtained from a group of 10 healthy controls (Rojkova et al., 2016). The analyses were focused on different language-related dorsal and ventral tracts, being these white matter pathways commonly affected in individuals with aphasia (Ivanova et al., 2016). Three ventral tracts were studied: (1) the IFOF connecting fronto-temporal regions, crossing from one lobe to the other through the extreme capsule; (2) the ILF that connects the posterior inferior, middle, and superior temporal gyri with the temporal pole; and (3) the UF which links the temporal pole with frontal areas (Catani and Thiebaut de Schotten, 2008). Three dorsal tracts were also explored, corresponding with the three segments of the AF: (1) the long segment that connects the frontal (including Broca’s area and the premotor cortex) and the temporal cortices (including Wernicke’s area and the middle and inferior temporal gyri); (2) the anterior segment that connects the same frontal areas with the angular and supramarginal gyri in the inferior parietal cortex; and (3) the posterior segment which connects the same parietal areas with the inferior and middle temporal gyri (Catani et al., 2005). Different measures were explored for each of the studied tracts. First, *Tractotron* provided the *probability* of a given tract to be affected by the brain lesion (≥ 50% was considered pathological) and the *percentage* of damage of each tract. Second, *Disconnectome maps* software provides a spatial map representing the probability of remote areas to be indirectly affected by the lesion. These indexes allowed to explore the remote impact of the focal brain lesions in the brain circuitry. Thus, the normalized lesion of subject P was used as seed point to identify which tracts passed through the lesion. Subject P’s disconnectome map was thresholded at a value of p > 0.9. A detailed description of these methods and software is reported in Foulon et al. (2018).

### Statistical Analyses

First, in order to evaluate longitudinal changes due to treatment effects, performance of subject P in each test included in the LNEs was either compared to the performance of the matched control group or to normative data. Specifically, for those tests that do not provide normative data for the age range of subject P, her performance was compared to the achievement of the control group on these tests. Notice that this served the purpose of the main aim of the study, that is to establish the effect of the different treatments on aphasia recovery, seeking for the return of subject P to an average performance in primary and secondary outcome measures. Statistical comparisons were performed using one-tailed Crawford’s modified *t*-tests (Crawford et al., 2010), as done in previous studies (François et al., 2016; Birba et al., 2017; Cervetto et al., 2018). This statistic allows the comparison between a single subject and a control group. It has proven to be robust for non-normal distributions, and it has low rates of type-I error. Effect sizes for all results are reported as point estimates (Z_CC_) (Crawford et al., 2010) (see **Table S1**). In all analyses, the alpha level was set at *p* < 0.05. For those tests reporting normative data, raw scores derived from subject P’s performance were standardized (percentile or decatype) (for details see **Tables 1** and **2**), unless specified otherwise.

**Table 1:** Language Assessment.

	Subject P				Reference value ^a^	
	LNE1	LNE2	LNE3	LNE4	Mean	SD
*Primary Outcome Measures*						
**Aphasia Quotient- Western Aphasia Battery Revised (WAB-R AQ)**	78.4*	92.6	95.8	94.2	95.13	2.73
Information Content	8*	10	10	10	10	0
Fluency	8*	10	10	10	10	0
Comprehension	8.5	9.7	9.9	10	9.01	0.78
Repetition	8	9	9.2	9	9.31	0.91
Naming	6.7*	7.6*	8.8	8.1	9.24	0.72
*Secondary Outcome Measures*						
**Snodgrass and Vanderwart Object Pictorial Set (SVOPS)**	138*	167*	220	211*	232.86	10.48
**Nombela 2.0 Semantic Battery (NSB)**						
Picture Naming	23*	39	51	53	44.29	9.64
Semantic Fluency	56*	79	102	79	136.71	35.4
Word-Picture Matching	29*	32	35	34	36.67	2.73
**Boston Naming Test (BNT)^1^**	23*	31*	52	40	46.41^b^	4.4^b^
**Peabody: Picture Vocabulary Test III (PPVT-III)^2^**	2	42	63	39	Standard scores^†^	
**Token Test (Shortened version) (TT-sv)^3^**	<5	95	50	70	Standard scores^†^	
**Psycholinguistic Assessments of Language** **Processing in Aphasia (PALPA)**						
Repetition: Nonwords (PALPA-8)	16*	22	24	23	23.42	0.79
Repetition: Imageability x Frequency (PALPA-9)	147*	160	–	–	160	0.00
Repetition: Sentences (PALPA-12)	26*	23*	28*	–	35.14	0.69
Reading: Visual Lexical Decision (PALPA-25)	138*	142*	138*	–	151.43	3.03
Reading: Grammatical Class (PALPA-32)	76*	75*	72*	–	80	0
Reading: Nonwords (PALPA-36)	20*	19*	19*	–	23.86	0.38
Reading: Sentences (PALPA-37)	33*	24*	32*	–	36	0
Semantics: Spoken Word-Picture Matching (PALPA-47)	36*	37	39	–	38.14	0.69
Semantics: Written Word-Picture Matching (PALPA-48)	35*	40*	38	–	38.28	0.76
Semantics: Spoken Word-Written Word Match (PALPA-52)	33*	32*	34	–	36.80	1.64
Semantics: Picture Naming (PALPA-53)	29*	34*	36	38	38.20	1.30
Semantics: Picture Naming x Frequency (PALPA-54)	53*	55*	55*	–	58.85	1.07
Spoken Sentence-Picture Matching (PALPA-55)	48*	52	53	53	56.28	2.69
Written Sentence-Picture Matching (PALPA-56)	46*	48*	53	52	55.14	2.54
**Controlled Oral Word Association Test (COWAT)**	9	23	15	17	24.14	7.97

Asterisks (*) indicate significant differences at p < 0.05. Statistical comparisons were performed using one-tailed Crawford’s t-tests in all cases except for tests marked with^†^. No statistical comparisons were needed for these tests since standardized scores provide a framework for comparing subject P’s performance against normative data. ^a^Reference values represent the mean scores and standard deviations (SD) of the control group, except for ^b^, that represents the normative mean scores and SDs provided by the test, and ^†^, that refers to normative data. ^†^The numbers in each evaluation are the percentiles corresponding to the raw score obtained by subject P. Underlined scores are bellow two SD of the normative mean. Normative data were obtained from: ^1^Halperin et al. (1989), ^2^Dunn et al. (2006), and ^3^Olabarrieta-Landa et al. (2017).

**Table 2:** Neuropsychological Assessment.

	Subject P				Reference value
	LNE1	LNE2	LNE3	LNE4	
*Executive Functions and Attention Tests*					
**Neuropsychological Evaluation of Executive Functions in Children** (**ENFEN)^1^**					
Grey trail	6	6	4	–	standard scores**^†^** (scale 1 to 10)
Color trail	3	4	4	–	
Interference	6	6	6	–	
**Stroop Test^2^**					
Word reading	< 5	< 5	< 5	–	standard scores**^‡^** (scale 1 to 99)
Colors naming	< 5	10	15	–	
**Five-Digit Test (FDT)^3^**					
Reading numbers	1	2	1	1	standard scores**^†^** (scale 1 to 10)
Counting	1	1	3	3	
Alternate	1	1	2	3	
Inhibition	8	9	9	10	
Flexibility	1	1	5	7	
**d2 Attention Test (d2 TESTS)^4^**					
Concentration	25	95	95	98	standard scores**^‡^** (scale 1 to 99)
Fluctuation	55	60	40	10	
Items processed	70	95	99	95	
Number of successes	25	96	99	98	
Omission errors	1	40	80	85	
Commission errors	25	35	60	85	
*Memory Tests*					
**Test of Memory and Learning (TOMAL)^5^**					
Verbal memory	75	99	–	–	standard scores**^‡^** (scale 1 to 99)
Non-verbal memory	95	99.6	–	–	
Composite memory	84	99.6	–	–	
Verbal delayed recall	37	91	–	–	
Attention and concentration	9	16	–	–	
Sequential memory	25	37	–	–	
Free recall	50	75	–	–	
Associative recall	2	37	–	–	
Learning	9	63	–	–	
**Digit Span^6^**					
Direct	10	10	10	–	standard scores**^‡^** (scale 1 to 99)
Inverse	50	50	50	–	
*Visuoconstructive and Visuoperceptive Functions*					
**Rey-Osterreith Complex Figure Test (ROCF)^7^**					
Copy	50	–	–	–	standard scores**^‡^** (scale 1 to 99)
Immediate recall	80	–	–	–	
**Benton Laboratory of Neuropsychology Tests (BLNT)^8^**					
Finger localization	49	–	–	–	53**^§^**
Visual form discrimination	41	–	–	–	23**^§^**
Judgment of line orientation	26	–	–	–	17**^§^**
Right-left orientation	18	–	–	–	16**^§^**

Reference data were obtained from either the test’s manual or published normative data as indicated by the reference of the superscript numerals located by each test’s name. ^1^Portellano et al. (2009). ^2^Rivera et al. (2017). **^3^**Sedó (2007). ^4^Brickenkamp and Cubero (2002). **^5^**Reynolds and Bigler (1996). ^6^Gardner (1981). ^7^Arango-Lasprilla et al. (2017). ^8^Rey et al. (1999). There are no children’s normative data for BLNT; reference values used are based on an adult sample. **^†^**Standard scores ranging from 1 to 10 (decatypes) have, by definition, a mean of 5.5 and a standard deviation of 2. **^‡^**Percentiles (standard score 1 to 99) have a median of 50. Underlined scores are bellow two standard deviations of the normative mean. **^§^**Test cut-off value; scores greater than the cut-off are considered within a normal range.

Second, results derived from each NE were analyzed in three different ways: i) in order to explore naming gains promoted by each INT, McNemar tests (two-tailed) were used to compare performance in the first NE after each INT against naming performance for those same words in NE0; ii) to track the evolution patterns of the potential gains found in NE1_1_ and NE2_1_, performance in each of the subsequent NEs (2-6) was compared to the performance in the first evaluation after INT (NE1_1_ and NE2_1_). The analyses i) and ii) were performed independently for the treated and untreated words; iii) performance in treated and untreated words in each NE was compared *via* Chi-squared tests (with Yate’s correction).

## Results

### Findings From Language and Neuropsychological Evaluation 1 (LNE1): Baseline

In relation to the primary outcome measures, subject P obtained a WAB-R AQ score of 78.4, which is significantly lower than the cut-off score for adults (≤ 93.8) and the mean of the age-matched control group (95.13 ± 2.73; Crawford’s t, one-tailed = -5.73; *p* ≤ 0.001). Her language deficits were characterized by impoverished information content with fluent yet anomic speech production. Specifically, significant lower scores were found in the subtests of the WAB-R targeting information content and fluency in spontaneous speech, and naming (see **Tables 1** and **S1**), whereas performance in comprehension and repetition did not differ from that of the control group. According to the WAB taxonomic criteria, this profile was compatible with an anomic aphasia (Kertesz, 1982). The mean WAB-R AQ of the control group (95.13) was above the cut-off score (93.8) for the clinical diagnosis of aphasia in adults (Kertesz, 1982). However, 3/7 control children obtained AQ scores slightly below the cut-off (92.4, 92.5, and 93.3). These children, being the youngest ones (8 years), committed a few failures in comprehension of reversible sentences in the sequential commands subtest of the WAB-R. Despite this age-dependent limitation, the WAB-R was considered appropriate for being administered to subject P.

Regarding secondary outcome measures, subject P’s scores were significantly lower than those of the control group in all selected naming measures (SVOPS, BNT, picture naming and semantic fluency [NSB], PALPA-53, and PALPA-54), auditory comprehension of nouns (word-picture matching [NSB], PALPA-47, and PALPA-52), auditory comprehension of sentences (PALPA-55), word repetition (PALPA-9), nonwords repetition (PALPA-8), sentence repetition (PALPA 12), and reading (PALPA-25, PALPA-32, PALPA-36, PALPA-37, PALPA-48, and PALPA-56). Also, performance in auditory word comprehension (PPVT-III) and of sentences (TT-sv) was lower than the normative data for her age. Lastly, performance of subject P in the verbal fluency test (COWAT) was not different from controls, although a trend toward significance was found (see **Tables 1** and **S1**).

In relation to other cognitive functions, compared to normative data subject P showed poor performance on most of the executive function tests (see **Table 2**), reflecting slow processing speed (word reading [STROOP]; number reading [FDT]), limited cognitive flexibility (color trails [ENFEN]; alternation and flexibility [FDT]) and low selective and sustained attention levels (concentration [d2 Test]). Yet, executive function impairments were not generalized, since subject P showed high scores in tests measuring inhibition of a prominent response (inhibition [FDT]). Furthermore, subject P’s performance on most of verbal and nonverbal memory tests was within the normal range of normative data (see **Table 2**) except for the reduced auditory-verbal short-term memory (Digit Span). The scores obtained by subject P in tests assessing visuoconstructive and visuoperceptive functions, which are mostly related to the undamaged right hemispheric functioning, were within the normal range (**Table 2**).

### Findings From Language and Neuropsychological Evaluation 2 (LNE2): DP Alone

Regarding primary outcome measures, the WAB-R AQ of subject P significantly improved after 12 weeks of DP treatment alone (see LNE2 column in **Table 1**). The AQ score increased 14.2 points (from 78.4 to 92.6) indicating that she could be considered a “good responder” to the pharmacological intervention (Berthier et al., 2009; Cherney et al., 2010). In fact, in the LNE2, the scores obtained in information content and fluency in spontaneous speech improved and they were comparable to the performance of the control group. Statistically significant lower scores were only found in the naming subtest of the WAB-R, which remained moderately impaired (**Tables 1** and **S1**).

In relation to secondary outcome measures, subject P showed improved naming abilities in both noun retrieval (picture naming and semantic fluency [NSB]), and auditory word (word-picture matching [NSB], PPVT-III, PALPA-47) and sentence (TT-sv, PALPA-55) comprehension. Likewise, improvements promoted by DP alone were found in words and nonwords repetition (PALPA-9 and PALPA-8). Performance in these tests was comparable to controls, and, in the case of the PPVT-III, fell within the normal range of the normative data. Comprehension improved slightly for written words (PALPA-48) and written sentences (PALPA 56), yet remained significantly lower than the performance of the control group. Conversely, sentences repetition (PALPA-12) showed a mild decrement (**Table 1**). As regard other cognitive functions (**Table 2**), high scores in selective and sustained attention were obtained (d2 Test) in comparison to normative data. Although an increase in processing speed was observed in some tests (word reading [STROOP], and in the number items of processed [d2 Test]), other measures of this domain remained low (e.g., reading numbers [FDT]). Further, slight improvements in cognitive flexibility were also found (color trails [ENFEN]), but this finding was not substantiated by other tests (alternate and flexibility [FDT]). Likewise, Digit Span remained low (**Table 2**).

### Findings From Language and Neuropsychological Evaluation 3 (LNE3): DP-INT1

This evaluation assessed gains in language after two weeks (weeks 12–14) of DP-INT1 and another 12 weeks of DP treatment. Concerning primary outcome measures, there were no statistically significant differences between subject P and the control group neither in the WAB-R AQ nor in the WAB-R subtests, meaning that subject P achieved an average performance on all language domains. This was the first evaluation in which she showed a naming score comparable to the controls (naming subtest of the WAB-R) (see **Table 1**).

Regarding secondary outcome measures, all improvements observed after DP treatment alone (LNE2) remained in LNE3 (as revealed by comparison of subject P’s performance with the control group’s). Besides, at this endpoint, further improvements were observed in almost all language-related secondary outcome measures: (a) noun retrieval test (SVOPS, BNT, picture naming, and semantic fluency [NSB], and PALPA-53); (b) all auditory word comprehension tests (word-picture matching [NSB], PPVT-III, PALPA-47, and PALPA-52); (c) auditory sentence comprehension (PALPA-55; but not in TT-sv); (d) nonword repetition (PALPA-8); (e) written-recognition of spoken words (PALPA-52) and comprehension of written sentences (PALPA-56). However, in the picture naming x frequency subtest (PALPA-54), subject P’s performance remained significantly lower than that of the control group, as well as sentence repetition and most of the reading measures. In relation to other cognitive functions, no relevant changes in measures of executive, attention or memory functions were observed at LNE3.

### Findings From Language and Neuropsychological Evaluation 4 (LNE4): Washout

In week 26, the dose of DP was gradually tapered off and suspended at week 30. As regards primary outcome measures, in LNE4 (week 34) it was observed that the improvements found in the primary outcome measures (WAB-R AQ and subtests) were maintained four weeks after withdrawal of DP (see **Table 1**). Thus, no statistically significant differences were found between subject P and the control group in any of the primary outcome measures at this point.

Concerning secondary outcome measures, the benefits observed in comprehension of auditory sentences (PALPA-55), nonword repetition (PALPA-8), and comprehension of written sentences (PALPA-56) remained unchanged. Performance in some naming tests dropped down (naming subtest of the WAB-R, BNT, and SVOPS), although only the SVOPS score was significantly lower than the control group. A slight decline in semantic fluency (NSB) and word comprehension (word-picture matching [NSB], and PPVT-III) was also observed, although performance did not differ from that of the control group (NSB) or was within the normal range (PPVT-III). In relation to other cognitive functions, compared to normative data, subject P maintained a within-average level of selective and sustained attention (d2 Test), cognitive flexibility (FDT) and attentional fluctuation (d2 Test) after drug withdrawal. Processing speed was within normal range when measured with the d2 Test, but was impaired when measured with the FDT.

### Naming Findings 1 (NE1)

**Figure 3:**
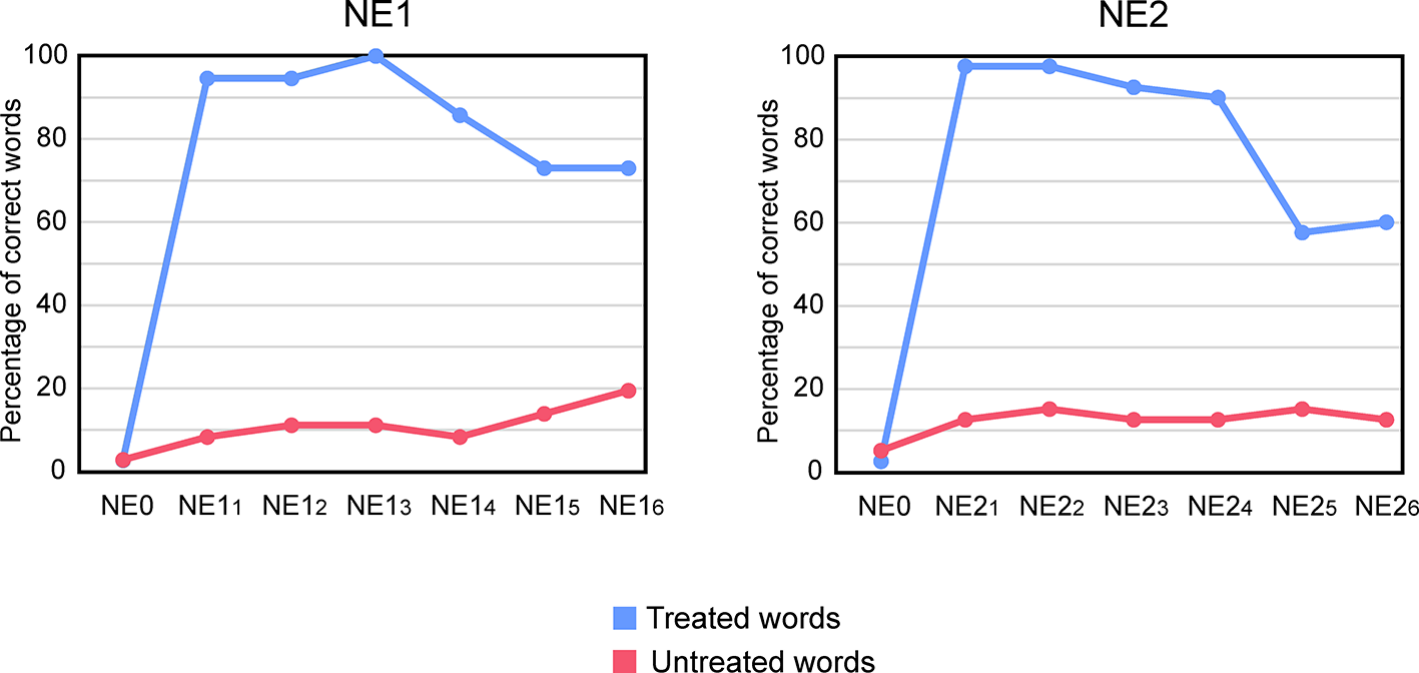
Performance of subject P in the multiple naming evaluations (NE). Percentage of correct words in each evaluation is shown. NE0 indicates the performance in the baseline NE performed before INT1. NE0 at the left of NE1_1-6_ indicates pre-treatment performance for the treated and untreated words used in INT1 and NE1_1-6._ NE0 at the left of NE2_1-6_ indicates pre-treatment performance for the treated and untreated words used in INT2 and NE2_1-6._ Six NEs were performed after INT1 (NE1_1-6_) and after INT2 (NE2_1-6_). NE1_1-6_ and NE2_1-6_ were performed: 20 min after the end of each INT (NE1_1_ and NE2_1_), and at days 2 (NE1_2_ and NE2_2_), 7 (NE1_3_ and NE2_3_), 21 (NE1_4_ and NE2_4_), 49 (NE1_5_ and NE2_5_), and 84 (NE1_6_ and NE2_6_) after the end of each INT.

**Figure 3** (left panel) depicts performance of subject P in NE0 (before INT1) and in the six NEs performed after INT1. Performance in NE0 was very low, with subject P naming correctly only 1/37 (2.70% correct) of the treated words and 1/36 (2.77% correct) of the untreated words.

At NE1_1_ subject P correctly named 35/37 of the treated words (95% correct). At this point, she produced two semantic paraphasias (“spinach” → *artichoke*; “rocking chair → *buck*) that were self-corrected immediately. Thus, naming performance was significantly higher in NE1_1_ compared to NE0 (McNemar, *p* ≤ 0.001). In NE1_2_, naming performance for treated words remained stable (35/37 [95% correct; McNemar, p = 1]) compared to NE1_1_. In this evaluation, errors consisted of an omission that was finally corrected with phonemic cueing, and a semantic paraphasia (“rocking chair” → *comfortable*), which was self-corrected. In NE1_3_ (7 days after the end of INT1), she correctly named 100% of treated words (37/37), being this not significantly different from the performance in NE1_1_ (McNemar, *p* = 0.500). At NE1_4_ (21 days after the completion of the INT1), there was a non-significant decrease in performance compared to NE1_1_ (30/37 [81% correct]; McNemar, *p* = 0.063). The decrease was due to 7 omissions. At NE1_5_ (after 49 day of the end of INT1), a significant decrease compared to NE1_1_ was observed (27/37 [73% correct]; McNemar, *p* = 0.008). Errors included 8 omissions and 2 semantic paraphasias (“asparagus” → *spinach*; “vertebra” → *pelvis*). Finally, at NE1_6_ (84 days after the end of the INT_1_), subject P showed the same level of performance than in NE1_5_ (27/37 [73% correct]) which was significantly lower than the performance in NE1_1_ (McNemar, *p* = 0.008). Thus, treatment-induced improvements in naming performance of treated words remained stable for 21 days (NE1_4_) while a significant decrement was detected in the evaluations performed after 49 days (NE1_5_ and NE1_6_). Nevertheless naming performance for treated words in all post-treatment evaluations (NE_1-6_) was significantly higher compared to baseline (NE0) (McNemar, *p* ≤ 0.001).

In relation to untreated words, performance in NE1_1_ did not significantly differ from performance in NE0 (McNemar, *p* = 0.500). Accordingly, naming performance in NE1_2-6_ was comparable to that on NE1_1_ (NE1_2_: McNemar, *p* = 1; NE1_3_: McNemar, *p* = 1; NE1_4_: McNemar, *p* = 1; NE1_5_: McNemar, *p* = 0.500; NE1_6_: McNemar, *p* = 0.125).

Finally, naming performance for treated and untreated words was comparable at baseline (NE0; χ^2^(1) = 0.44 p = 0.508) but higher for treated than for untreated words in all post-treatment NEs, revealing no generalization from treated to untreated words just after INT. Differences were statistically significant for: NE1_1_ (χ^2^(1) = 51.00, p ≤ 0.001), NE1_2_ (χ2(1) = 48.79, p ≤ 0.001), NE1_3_ (χ2(1) = 55.00, p ≤ 0.001), NE1_4_ (χ2(1) = 36.10, p ≤ 0.001), NE1_5_ (χ2(1) = 23.53, p ≤ 0.001), NE1_6_ (χ2(1) = 18.92, p ≤ 0.001).

### Naming Findings 2 (NE2)

**Figure 3** (right panel) depicts performance of subject P in NE0 and in the six NEs performed after INT2. Baseline (NE0) naming performance was 1/40 (2.5% correct) for treated words and of 2/40 (5% correct) for untreated words.

At NE2_1_ subject P produced 39/40 correct responses (97% correct) in treated words, committing a phonemic paraphasia. Thus, compared to NE0, naming performance for treated words significantly increased after treatment (McNemar, *p* ≤ 0.001). These results remained stable at NE2_2_ (treated words: 39/40 [97% correct; McNemar, *p* = 1], wherein she committed just an omission. At NE2_3_, she correctly named 37/40 [92% correct] of the treated words, which was not significantly different from the performance in NE2_1_ (McNemar, *p* = 0.500). At this timepoint, subject P produced one omission and two phonemic paraphasias. At NE2_4_ the number of correct responses in treated words was of 36/40 [90% correct] (4 omissions), comparable to the performance observed in NE2_1_ (McNemar, *p* = 0.250). At NE2_5_, a decrease in correct responses for treated words was observed (23/40 [57% correct]; 16 omissions and 1 semantic paraphasia) compared to NE2_1_ (McNemar, *p* ≤ 0.001). Finally, performance at NE2_6_ remained stable compared to NE2_5_ (23/40 [57% correct]; 15 omissions and 2 semantic paraphasias), which was significantly lower than the performance in NE2_1_ (McNemar, *p* ≤ 0.001). Thus, like in INT1, treatment-induced gains in naming remained stable for 21 days (NE2_4_), but a significant decrease was detected in the evaluations performed after 49 days (NE2_5_ and NE2_6_).

In relation to untreated words, performance in NE2_1_ did not significantly differ from performance in NE0 (McNemar, *p* = 0.250). Naming performance in NE2_2-6_ was comparable to that on NE2_1_ (for all comparisons: McNemar, *p* = 1).

Finally, naming performance was significantly higher for treated words than for untreated ones in all NEs, but N0, revealing no generalization from treated to untreated words: NE0 (χ^2^(1) = 0.35, p = 1), NE2_1_ (χ^2^(1) = 55.00, p ≤ 0.001), NE2_2_ (χ^2^(1) = 52.01, p ≤ 0.001), NE2_3_ (χ^2^(1) = 48.17, p ≤ 0.001), NE2_4_ (χ^2^(1) = 45.03, p ≤ 0.001), NE2_5_ (χ^2^(1) = 13.85, p ≤ 0.001), NE2_6_ (χ^2^(1) = 15.88, p ≤ 0.001).

### Neuroimaging Findings

#### Lesion Location

**Figure 4:**
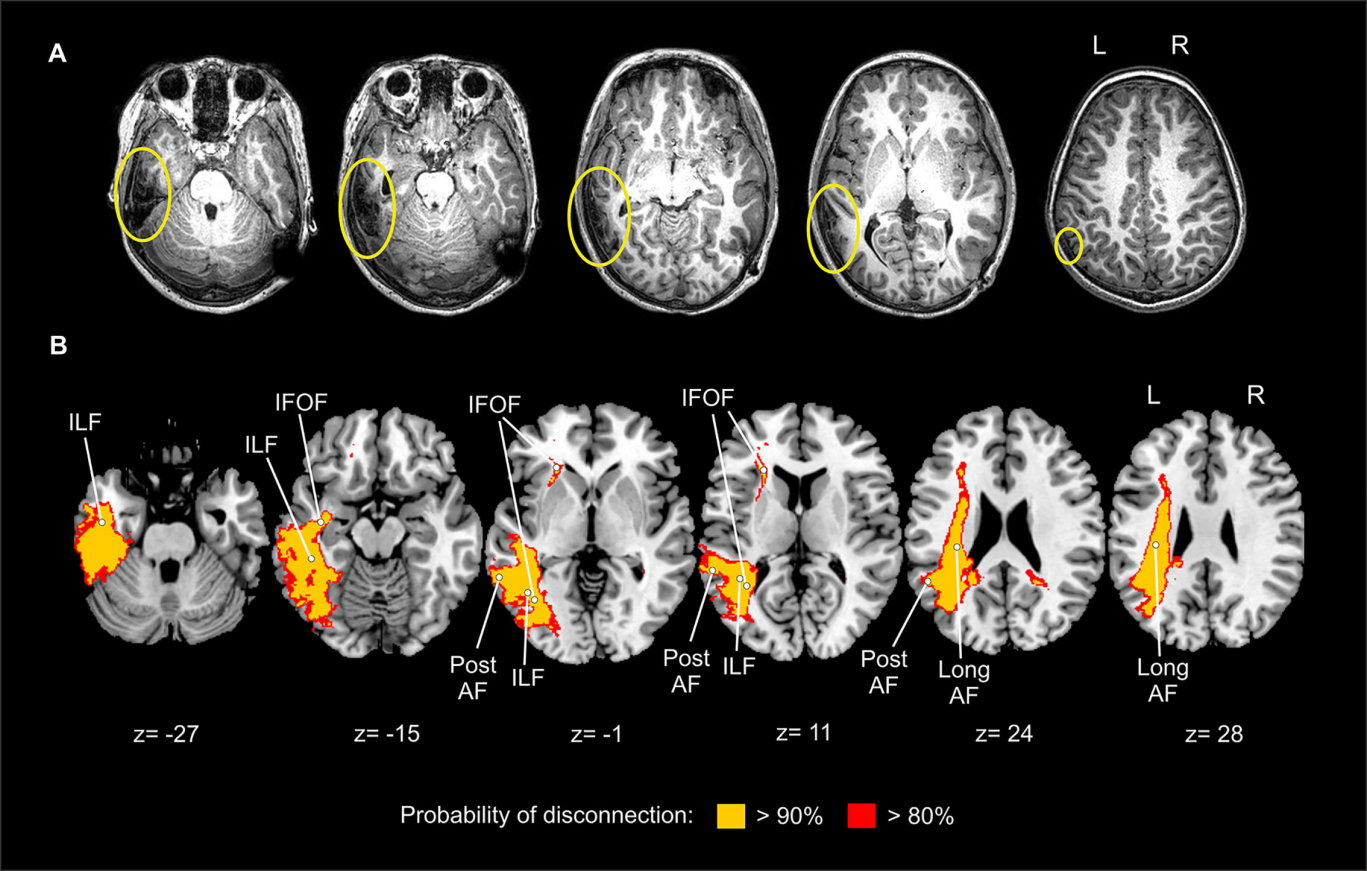
Depiction of the brain lesion and disconnection pattern. **(A)** MRI T_1_-weighted image showing the lesion in the left temporo-parietal cortex in native space. Yellow circles indicate brain lesions. **(B)** Probability of disconnection of brain areas not directly affected by the lesion as revealed by the *Disconnectome map* software. Two different probability thresholds are presented. The disconnection map is overimposed on a brain template in standard MNI space. L, left; R, right; ILF, inferior longitudinal fasciculus; IFOF, inferior fronto-occipital fasciculus; post AF, arcuate fasciculus posterior segment; long AF, arcuate fasciculus long segment.

MRI performed 6 months after the TBI showed left cortical tissue damage, mostly involving the inferior temporal gyrus (Brodmann area [BA] 37) and to a lesser extent the middle temporal gyrus (BA21) and the angular gyrus (BA39) (**Figure 4A**). There was also a focal cortical atrophy and gliosis in the subcortical white matter, causing a discrete retraction of the temporal horn of the left lateral ventricle. The ventricular-peritoneal shunt was correctly placed in the occipital horn of the right lateral ventricle.

#### Mapping Disconnection: Tractotron and Disconnectome Maps

Tractotron revealed that in the left hemisphere, the AF anterior segment showed a probability of 48% to be directly affected by the lesion; and the AF long and posterior segments showed a probability of 98%. Ventrally, the ILF showed a 100% probability of involvement; the IFOF showed a probability of 92%, while the UF was unlikely to be affected (probability of 0%). In the right hemisphere, none of these tracts were damaged (all probabilities were equal to 0%). The high probability of affectation found for the AF posterior and long segments, the ILF and the IFOF are in line with the cortical damage observed in subject P, which affected the inferior and middle temporal gyri and the angular gyrus, regions connected by these tracts (Catani and Thiebaut de Schotten, 2008).

Yet, the probability of a given tract to be affected does not inform on the amount of damage. To obtain this measure, the proportion of damage was extracted. The proportions of each tract to be affected by the lesion were: AF anterior segment: 0%; AF long segment: 29%; AF posterior segment: 32%; ILF: 13%; IFOF: 4%; UF: 0%. Finally, note that those distant cortical areas that showed a high probability of disconnection (> 80%) due to the TBI are in fact the ones connected by the affected white matter pathways as revealed by Tractotron (**Figure 4B**).

## Discussion

In the present intervention study, we described the case of subject P, a girl with chronic anomic aphasia secondary to a TBI in the left temporo-parietal region. She received three successive treatments: (1) DP alone; (2) a combination of DP and INT; and (3) INT alone. Multiple language and other cognitive domains evaluations were performed at baseline and at different time-points (**Figure 2**) in order to track changes promoted by these interventions. Results obtained from these evaluations were compared to a socio-demographically matched control group.

Several important findings of our study should be highlighted. First, at baseline, subject P showed significantly lower scores than the control group in the primary and secondary outcome measures targeting language, attentional, and executive functions. Second, treatment with DP alone (week 0 to week 12) induced improvements in primary outcome measures (see LNE2 results). Aphasia severity and scores in different language domains (fluency and information content during spontaneous speech and naming) improved, and at this point subject P performance was comparable to the control group’s in all primary outcome measures (WAB-R AQ and WAB’s subtests), except for naming. Third, combined treatment with DP-INT1 (week 12 to week 26, see LNE3 results) further increased the WAB-R AQ, placing the language deficits of subject P in the non-aphasic range. Fourth, the combined intervention provided further gains in picture naming, the most affected language function at baseline (LNE1). Fifth, secondary outcome measures improved with DP alone (LNE2), denoting the beneficial effect of the drug, and most of the differences to the controls observed at baseline disappeared with combined treatment (DP-INT1; LNE3). Lastly, most gains provided by DP intervention were stable 4 weeks after withdrawal. It is noteworthy that at washout evaluation, the WAB-R AQ remained within the normal range as compared to the control group.

Language disorders in childhood often have important implications in everyday life and represent a risk factor of developing anxiety and social problems in adulthood (Brownlie et al., 2016; Ryan et al., 2018). Currently, the only available treatment for CA is speech-language therapy, and although it often promotes recovery of linguistic and other cognitive functions, restoration is far for being complete. However, research aimed at finding new therapeutic strategies to improve outcomes in CA is still underdeveloped. Overlooking the investigation of new therapeutic approaches to improve CA may have negative consequences such as preventing the development of language and communication skills during childhood and adolescence. There is now encouraging evidence derived from model-based interventions indicating that adult aphasia outcomes can be improved with intensive aphasia therapy and other therapeutic approaches (pharmacotherapy, non-invasive brain stimulation) (Pulvermüller and Berthier, 2008; Berthier and Pulvermüller, 2011; Breitenstein et al., 2017; Fridriksson et al., 2018). Taking advantage from data on these new interventions in adults with aphasia, in the present case study we used a similar therapeutic approach demonstrating, for the first time that DP is safe and well tolerated in CA and can be used alone and in combination with a tailor-made aphasia therapy (e.g., INT) to boost recovery of language and cognitive deficits.

### Pre-Treatment Behavioral Profile and Brain-Behavior Relationships

Baseline testing (LNE1) with the WAB-R classified the language disorder in subject P as anomic aphasia (Kertesz, 1982), yet she also displayed deficits in phonology and semantic processing. On the WAB-R, the deficits were mainly observed in spontaneous speech (information content and fluency), and naming. Furthermore, subject P showed significantly lower scores in most of the secondary outcome measures than the control group (auditory and visual-verbal comprehension, repetition, noun naming, and reading). Subject P also showed low performance in tests measuring executive functions, attention, and auditory verbal short-term memory, manifested by slow processing speed, limited cognitive flexibility, low selective and sustained attention levels, and reduced verbal span. Impairments in executive and attentional functions are common after TBI due to diffuse cerebral damage that frequently affects the white matter bundles in frontal and temporal lobes (Levin, 2003; Vik et al., 2006). Although memory dysfunction is usually associated to oral language deficits in children with TBI (Conde-Guzón et al., 2009), the performance of subject P on the different memory subscales revealed that this function was preserved, except for a reduced digit span. In addition, performance in visuoconstructive and visuoperceptive tests was preserved.

Structural MRI in the chronic stage showed a large contusion in the left temporo-parietal cortex together with focal cortical atrophy and gliosis in the subcortical white matter. Our lesion-based approach suggests that the tract with major proportion of damage was the AF posterior segment, which connects regions that were specifically damaged in subject P (i.e., inferior parietal cortex and ventral posterior temporal cortex). This segment is part of the indirect connection of the AF system implicated in verbal repetition (Forkel et al., 2020) and reading (Thiebaut de Schotten et al., 2014). Notice that at baseline evaluation (LNE1, **Table 1**), both nonword (PALPA-8) and sentence (PALPA-12) repetition, as well as reading (PALPA-25, PALPA-32, PALPA-36, PALPA-37) were impaired. Ventrally, the ILF was the tract with the major proportion of damage. This tract runs in parallel from posterior to anterior parts of the temporal lobe and is implicated in lexical access (Herbet et al., 2019). This is consistent with the fact that naming was the main deficit of subject P. Thus, the high probability of affectation of these tracts together with the observed cortical involvement of temporo-parietal areas (BA21, BA37, and BA39) may explain the prominent naming difficulties found in subject P, as well as the pattern of committed errors (semantic paraphasias). For instance, axonal degeneration of the ILF is related to naming deficits and the production of semantic paraphasias in post-stroke aphasia (McKinnon et al., 2018) and in patients with brain tumors (Sierpowska et al., 2019). In addition, the ILF has been systematically implicated in semantic processing, lexical access (Nugiel et al., 2016; Herbet et al., 2019) and word learning involving lexical-semantic association in healthy subjects (Ripollés et al., 2017). In this line, BA37, which is damaged in subject P, is an important cortical hub for two distinct networks implicated in visual recognition (perception) and semantic functions (Ardila et al., 2015), and its damage is associated with fluency, comprehension, repetition, and naming impairments after stroke (Gleichgerrcht et al., 2015). The subtle involvement of BA21 in the posterior middle temporal cortex may have altered semantic control for comprehension (Noonan et al., 2013). Finally, despite the small size of the parietal cortical damage, the angular as well as the supramarginal gyri may be disconnected due to the affectation of the AF posterior segment, as revealed by the lesion analyses. Therefore, although it seems that the lesion sizes were not large enough to induce major disconnections, they were strategically placed to interrupt intrinsic connections within the left perisylvian language area. Unfortunately, since we only were able to perform a MRI study at baseline^2^, we could not compare pre- and post- treatment MRIs to explore the brain correlates of the observed improvements in naming.

### Drug Treatment Alone Improves Language and Cognitive Deficits

Although the beneficial action of the cholinesterase inhibitor DP is controversial in adult TBI, with studies showing both positive effects and lack of benefits (Walker et al., 2004; Warden et al., 2006; Shaw et al., 2013), our findings clearly show that CA may be improved with cholinergic potentiation. After 12 weeks of DP treatment (LNE2), a decrease in aphasia severity was found in subject P, as revealed by increased scores on both the WAB-R AQ and its subtests, except for naming. In fact, improvements were found for some naming tests (NSB subscale), but not for others (SVOPS, BNT, EPLA-53, EPLA-54). The treatment with DP alone also induced significant improvements in measures of verbal fluency (semantic and phonological), auditory-verbal comprehension (words and sentences), and word and nonword repetition. These linguistic improvements may be associated with enhancement of selective and sustained attention which eventually favored phonological and lexical processing for these stimuli. This is consistent with the role of anticholinesterase drugs, like DP, in improving sustained attention (Spiridigliozzi et al., 2007) and language function (Heller et al., 2004) in children. The bulk of the lesion in subject P was in the left temporal cortex, and this lobe contains more choline acetyltransferase than its homologous counterpart (Amaducci et al., 1981; Hutsler and Gazzaniga, 1996). Therefore, a neurobiological explanation for this finding would be that the language improvements could be accounted for cholinergic-induced neural plasticity in left perilesional temporal cortical areas and white matter tracts (ILF and IFOF), though the contribution of remote right cortical regions cannot be dismissed.

The gains produced by DP in selective and sustained attention were not associated with improvement in other frontal executive functions, which most likely resulted from diffuse axonal injury and the pressure effects of acute hydrocephalus on frontal tissue. Although an increase in processing speed was observed in d2 Tests, the performance on other tests evaluating this domain remained low. Likewise, there were slight improvements in cognitive flexibility (ENFEN), but this finding was not substantiated by other tests. Repetition and written comprehension of sentences, reading functions, and auditory-verbal short-term memory (Digit Span) also remained altered. This is in accord with Martin and Ayala’s findings (2004) who have reported significant correlations between the severity of language impairment (in both phonological and lexical-semantic measures) and the size of digit and word span in individuals with aphasia.

Reading problems persisted in subject P. The fact that associative visual areas in the left inferior occipito-temporal cortex, such as the visual word form area (VWFA), were damaged, might be the simplest explanation of the reading deficits in subject P. The VWFA is a region specifically devoted to the recognition of the written words in literate persons (Cohen et al., 2000; López-Barroso et al., 2020) and its damage causes alexia. Although compensation by recruitment of the VWFA homolog in the right hemisphere can take place (Cohen et al., 2003), this plastic shift may require intensive training.

### Combined Therapy Increases Gains Obtained With Drug Monotherapy

Treatment with DP alone improved language deficits in subject P. Nevertheless, recent evidence suggests that cholinergic stimulation in adults with TBI is useful when combined with environmental enrichment (De la Tremblaye et al., 2019). The current findings further support the importance of augmenting the effect of DP on brain tissue with INT. Almost all scores obtained under treatment with DP alone showed further improvements after two weeks of combination therapy (LNE3). Moreover, in comparison with baseline (LNE1) and the evaluation after DP alone (LNE2), the highest gains after combination therapy (LNE3) were in several measures of naming production (see **Table 1**). Naming evaluations post-INT1 (NE1) under ongoing DP treatment (weeks 14-26) showed significantly better performance for treated items than for untreated ones. During this time period, gains in treated items were maintained, whereas low scores in untreated items remained unchanged.

Since then, DP was gradually tapered off (weeks 26-30) and followed by a washout period (weeks 30-34) and a new phase of INT (INT2). Post-washout evaluation (LNE4) showed that improvements observed in the WAB-R AQ decreased slightly but remained comparable to the scores of the control sample and well above subject P’s baseline score. At this point, the score on the naming subtest of the WAB-R presented a slight decrease, but decrements were more evident in other naming tasks (SVOPS, semantic fluency, BNT). By contrast, the benefits observed in other language tasks (fluency, word and nonword repetition, auditory sentence comprehension, sentence reading comprehension, and phonological and semantic fluency) were stable. Likewise, washout testing revealed that subject P maintained an above-average level in several attentional and cognitive flexibility measures. Naming evaluations post-INT2 alone (NE2) showed a similar tendency to the outcomes of naming evaluation in NE1 except for the more pronounced decline in the two last NE2s.

Two of these results were unexpected. First, although beneficial effects of DP-INT1 were generalized to several language and cognitive domains, it was surprising that untreated items showed no improvement. The lack of generalization did not result from differences in selection of treated and untreated words, because both sets of words were closely matched controlling key linguistic variables. Although this negative evidence deserves further research, our results suggest that the effect of the DP on untreated nouns was not as powerful as when the drug was combined with intensive noun training, aimed to strengthen experience-dependent plasticity. Similarly, combined dexamphetamine with naming therapy in two subjects with chronic adult post-stroke aphasia improved treated nouns but not untreated ones, nor a control nonword reading task (Whiting et al., 2007). The second unexpected finding was that the results of post-INT2 (NE2) evaluations (without pharmacotherapy) were similar to those obtained in post-INT1 evaluations (NE1) while subject P was still under DP treatment. A likely explanation could be that the previous prolonged treatment with DP (duration: 26 weeks) induced long-lasting brain changes that were then profitable seized by the application of INT2 after a short washout period (4 weeks). Thus, a lesson to be learned from this finding would be that once the brain has been primed with a combined intervention (DP-INT1), it would be similarly responsive to a single modality of intervention (INT2 or a drug) applied at a later stage (see Berthier et al., 2009; Berthier, 2020).

Finally, the results of the present study should be interpreted considering some limitations. First, this is an open-label study performed in a single subject. Thus, randomized controlled trials in larger samples are strongly needed. Second, we initiated the drug treatment before aphasia therapy, so that the effect of the naming training alone could only be evaluated after previous treatment with DP. Therefore, other designs should be evaluated in the future. Lastly, it is not possible to rule out that some beneficial changes in subject P may have resulted from the continued maturation and evolution of cognitive and language processes that may be partially blended with the beneficial effects of the two therapeutic interventions. Yet, this is unlikely, at least for naming ability, since no improvements were seen for untreated items which served as control. A further strategy to reduce the confounding factor of language and cognitive development and maturation in outcomes of an intervention trial in CA is performing multiple baseline assessments. Multiple baseline assessments were not used in this study. Notice that we performed a very comprehensive language and cognitive evaluation that took several days to be completed. This may preclude the utilization of multiple baseline testing. Indeed, longer and repetitive evaluations are very tiring, particularly for children, and may reduce motivation, putting at risk adherence to evaluation and treatment. The rationale to use such a large test battery in subject P was to examine, for the first time, the effect of DP and INT not only in language functions but also in several other cognitive domains, which are commonly affected after TBI and may influence outcomes. To overcome this limitation, future studies may perform multiple baseline assessments in the most affected language domain(s) (e.g., naming in subject P) or in the domain(s) targeted for the intervention.

In summary, subject P, who presented an acquired aphasia after suffering a TBI involving the left temporo-parietal region, significantly improved anomia and related cognitive deficits through the use of a cholinergic agent (DP) alone and in combination with INT.

## Data Availability Statement

The dataset that support the findings of this study will be available upon request from the corresponding author.

## Ethics Statement

The Ethics Research Committee *Provincial de Málaga* approved this study.

## Author Contributions

All authors contributed to the article and approved the submitted version. GD, MM, MT-P, MB, LE, and DL-B were involved in conception and design, acquisition of data, or analysis and interpretation of data. GD, MM, MT-P, LM-C, LE, and DL-B were involved in cognitive and language assessment. GD, MT-P, MB, and DL-B interpreted neuroimaging data. GD, MT-P, MB, and DL-B drafted the article and revised it critically for important intellectual content.

## Funding

MM has been funded by I Plan for Research and Transfer of the University of Malaga (Introduction to Research Scholarship for Undergraduate and Master’s Students, 107/2018). LM-C has been supported by funds from the European Social Fund (E-29-201-0705972). LE has been funded by a PhD scholarship from the Spanish Ministry of Education, Culture, and Sport under the FPU program (FPU17/04136). MT-P has been funded by a postdoctoral fellowship from the University of Malaga. DL-B has been supported by a I+D+i Project, Andalucia and European Union Funds (FEDER) (UMA18-FEDERJA-221). The work was supported in part by the Ministerio de Economía, Industria y Competitividad, Instituto de Salud Carlos III, Madrid, Spain under Grant: PI16/01514.

## Conflict of Interest

MB has received honoraria from Pfizer, Eisai, Janssen, Novartis, Lundbeck, TEVA and Nutricia, and consultancy fees from Merz, Eli Lilly, and GlaxoSmithKline.

The remaining authors declare that the research was conducted in the absence of any commercial or financial relationships that could be construed as a potential conflict of interest.

## Footnotes

1 Intensive Naming Therapy 2 (INT2) was planned at the starting point of the trial. However, it was not intended to make direct comparisons between treatment with DP alone and DP-INT combined against INT alone. In fact, this new course of INT after the washout period was requested by the Ethical Committee and the parents of subject P. Thus, INT2 served the purpose of maintaining the benefits of the interventions on word retrieval deficits to further support the patient needs.

2 Multimodal MRI studies at baseline and repeated scanning at different time points were not performed because exposing patients with ventricular shunts to prolonged 3-Tesla MRI procedures poses a significant risk of unintentional changes in shunt settings. Therefore, subject P only underwent a single, rapid acquisition of structural MRI. Despite this, reprogramming of the shunt was needed after the study.

## References

[B1] Amaducci L. Sorbi S. Albanese A. Gainotti G. (1981). Choline acetyltransferase (CHAT) activity differs in right and left human temporal lobes. Neurology 31, 799–805. 10.1212/WNL.31.7.799 7195501

[B2] Anderson V. Catroppa C. (2006). Advances in Postacute Rehabilitation after Childhood Acquired Brain Injury. Am. J. Phys. Med. Rehab. 85, 767–778. 10.1097/01.phm.0000233176.08480.22 16924189

[B3] Anderson V. Catroppa C. Haritou F. Morse S. Pentland L. Rosenfeld (2001). Predictors of Acute Child and Family Outcome following Traumatic Brain Injury in Children. Pediatr. Neurosc. 34, 138–148. 10.1159/000056009 11359102

[B4] Anderson V. Catroppa C. Morse S. Haritou F. Rosenfeld J. (2005). Functional Plasticity or Vulnerability After Early Brain Injury? Pediatrics 116 (6), 1374–1382. 10.1542/peds.2004-1728 16322161

[B5] Anderson V. Spencer-Smith M. Leventer R. Coleman L. Anderson P. Williams J. (2009). Childhood brain insult: can age at insult help us predict outcome? Brain 132, 45–56. 10.1093/brain/awn293 19168454

[B6] Aram D. M. Ekelmann B. L. Rose D. F. Whitaker H. A. (1985). Verbal and cognitive sequelae following unilateral lesions acquired in early childhood. J. Clin. Exp. Neuropsychol. 7, 55–78. 10.1080/01688638508401242 3980681

[B7] Aram D. M. (1998). “Acquired Aphasia in Children,” in Acquired Aphasia, 3rd ed. Ed. Sarno M. (California, USA: Academic Press), 451–480.

[B8] Arango-Lasprilla J. C. Rivera D. Ertl M. M. Muñoz Mancilla J. M. García-Guerrero C. E. Rodriguez-Irizarry W. (2017). Rey–Osterrieth Complex Figure–copy and immediate recall (3 minutes): Normative data for Spanish-speaking pediatric populations. NeuroRehabilitation 41 (3), 593–603. 28885225 10.3233/NRE-172241

[B9] Ardila A. Bernal B. Rosselli M. (2015). Language and visual perception associations: meta-analytic connectivity modeling of Brodmann area 37. Behav. Neurol. 2015:565871. 10.1155/2015/565871 25648869 PMC4306224

[B10] Asaridou S. S. Demir-Lira Ö.E. Goldin-Meadow S. Levine S. C. Small S. L. (2020). Language development and brain reorganization in a child born without the left hemisphere. Cortex 127, 290–312. 10.1016/j.cortex.2020.02.006 32259667 PMC8025291

[B11] Avila L. Riesgo R. Pedroso F. Goldani M. Danesi M. Ranzan J. (2010). Language and Focal Brain Lesion in Childhood. J. Child Neuropsychol. 25 (7), 829–833. 10.1177/0883073809350724 20110218

[B12] Babikian T. Asarnow R. (2009). Neurocognitive Outcomes and Recovery after Pediatric TBI: Meta-Analytic Review of the Literature. Neuropsych 23 (3), 283–296. 10.1037/a0015268 PMC4064005 19413443

[B13] Banaschewski T. Roessner V. Dittmann R. W. Santosh P. J. Rothenberger A. (2004). Non-stimulant medications in the treatment of ADHD. Eur. Child Adolesc. Psychiatry 13 Suppl 1, I102–I116. 10.1007/s00787-004-1010-x 15322961

[B14] Beitchman J. H. Wilson B. Johnson C. J. Atkinson L. Young A. Adlaf E. (2001). Fourteen-year follow-up of speech/language-impaired and control children: Psychiatric outcome. J. Am. Acad. Child Adolesc. Psychiatry 40 (1), 75–82. 10.1097/00004583-200101000-00019 11195567

[B15] Benton A. L. Sivan A. B. Hamsher K. Varney N. R. Spreen O. (1994). Contributions to Neuropsychological Assessment: A Clinical Manual (New York: Oxford University Press).

[B16] Berthier M. L. Pulvermüller F. (2011). Neuroscience insights improve neurorehabilitation of poststroke aphasia. Nat. Rev. Neurol. 7 (2), 86–97. 10.1038/nrneurol.2010.201 21297651

[B17] Berthier M. L. Hinojosa J. Martín M. C. Fernández I. (2003). Open-label study of donepezil in chronic poststroke aphasia. Neurology 60 (7), 1218–1219. 10.1212/01.wnl.0000055871.82308.41 12682346

[B18] Berthier M. L. Green C. Higueras C. Fernández I. Hinojosa J. Martín M. C. (2006). A randomized, placebo-controlled study of donepezil in poststroke aphasia. Neurology 67 (9), 1687–1689. 10.1212/01.wnl.0000242626.69666.e2 17101908

[B19] Berthier M. L. Green C. Lara J. P. Higueras C. Barbancho M. A. Dávila G. (2009). Memantine and constraint-induced aphasia therapy in chronic poststroke aphasia. Ann. Neurol. 65, 577–585. 10.1002/ana.21597 19475666

[B20] Berthier M. L. Pulvermüller F. Dávila G. Casares N. G. Gutiérrez A. (2011). Drug therapy of post-stroke aphasia: a review of current evidence. Neuropsychol. Rev. 21 (3), 302–317. 10.1007/s11065-011-9177-7 21845354

[B21] Berthier M. L. Dávila G. Green-Heredia C. Moreno-Torres I. Juárez y Ruiz de Mier R. De-Torres I. (2014). Massed sentence repetition training can augment and speed up recovery of speech production deficits in patients with chronic conduction aphasia receiving donepezil treatment. Aphasiology 28 (2), 188–218. 10.1080/02687038.2013.861057

[B22] Berthier M. L. De-Torres I. Paredes-Pacheco J. Roé-Vellvé N. Thurnhofer-Hemsi K. Torres-Prioris M. J. (2017). Cholinergic potentiation and audiovisual repetition-imitation therapy improve speech production and communication deficits in a person with crossed aphasia by inducing structural plasticity in white matter tracts. Front. Hum. Neurosci. 14:304 (11):304. 10.3389/fnhum.2017.00304 PMC5470532 28659776

[B23] Berthier M. L. (1999). Transcortical aphasias (UK: Psychology Press).

[B24] Berthier M. L. (2020). Ten key reasons for continuing research on pharmacotherapy for post-stroke aphasia. Aphasiology. 10.1080/02687038.2020.1769987

[B25] Best W. Greenwood A. Grassly J. Herbert R. Hickin J. Howard D. (2013). Aphasia Rehabilitation: Does Generalisation From Anomia Therapy Occur and Is It Predictable? A Case Series Study. Cortex 49 (9), 2345–2357. 10.1016/j.cortex.2013.01.005 23608067

[B26] Biederman J. Spencer T. (2000). Non-stimulant treatments for ADHD. Eur. Child Adolesc. Psychiatry 9 (Suppl 1), I51–I59. 10.1007/s007870070019 11140780

[B27] Birba A. Hesse E. Sedeño L. Mikulan E. P. García M. del C. (2017). Enhanced Working Memory Binding by Direct Electrical Stimulation of the Parietal Cortex. Front. Aging Neurosci. 9:178:178. 10.3389/fnagi.2017.00178 28642698 PMC5462969

[B28] Birkenkamp R. Zillmer E. (1998). The d2 Test of Attention (Goettingen: Hogrefe).

[B29] Borkowski J. G. Benton A. L. Spreen O. (1967). Word fluency and brain damage. Neuropsychologia 5, 135–140. 10.1016/0028-3932(67)90015-2

[B30] Bowen J. M. (2005). Classroom interventions for students with traumatic brain injuries. Prev. School. Failure. 49, 34–41. 10.3200/PSFL.49.4.34-41

[B31] Brauer J. Anwander A. Friederici A. D. (2011). Neuroanatomical prerequisites for language functions in the maturing brain. Cereb. Cortex. 21 (2), 459–466. 10.1093/cercor/bhq108 20566580

[B32] Breitenstein C. Grewe T. Flöel A. Ziegler W. Springer L. Martus P. (2017). Intensive speech and language therapy in patients with chronic aphasia after stroke: a randomised, open-label, blinded-endpoint, controlled trial in a health-care setting. Lancet 389 (10078), 1528–1538. 10.1016/S0140-6736(17)30067-3 28256356

[B33] Brickenkamp R. Cubero N. S. (2002). D2: test de atención. (Tea).

[B34] Brownlie E. B. Bao L. Beitchman J. (2016). Childhood language disorder and social anxiety in early adulthood. J. Abnorm. Child. Psych. 44 (6), 1061–1070. 10.1007/s10802-015-0097-5 26530522

[B35] Buckley A. W. Sassower K. Rodriguez A. J. Jennison K. Wingert K. Buckley J. (2011). An open label trial of donepezil for enhancement of rapid eye movement sleep in young children with autism spectrum disorders. J. Child Adolesc. Psychopharmacol. 21 (4), 353–357. 10.1089/cap.2010.0121 21851192 PMC3157749

[B36] Castellino S. M. Tooze J. A. Flowers L. Hill D. F. McMullen K. P. Shaw E. G. (2012). Toxicity and efficacy of the acetylcholinesterase (AChe) inhibitor donepezil in childhood brain tumor survivors: a pilot study. Pediatr. Blood Cancer. 59 (3), 540–547. 10.1002/pbc.24078 22238217 PMC3345166

[B37] Castellino S. M. Ullrich N. J. Whelen M. J. Lange B. J. (2014). Developing interventions for cancer-related cognitive dysfunction in childhood cancer survivors. J. Natl. Cancer. Inst. 106 (8). 10.1093/jnci/dju186 PMC4155432 25080574

[B38] Catani M. Thiebaut De Schotten M. T. (2008). A diffusion tensor imaging tractography atlas for virtual in vivo dissections. Cortex 44 (8), 1105–1132. 10.1016/j.cortex.2008.05.004 18619589

[B39] Catani M. Jones D. K. Fytche D. H. (2005). Perisylvian language networks of the human brain. Ann. Neurol. 57 (1), 8–16. 10.1002/ana.20319 15597383

[B40] Cervetto S. Abrevaya S. Martorell Caro M. Kozono G. Muñoz E. Ferrari J. (2018). Action Semantics at the Bottom of the Brain: Insights From Dysplastic Cerebellar Gangliocytoma. Front. Psychol. 9:1194:1194. 10.3389/fpsyg.2018.01194 30050490 PMC6052139

[B41] Chapman S. S. Ewing C. B. Mozzoni M. P. (2005). Precision teaching and fluency training across cognitive, physical, and academic tasks in children with traumatic brain injury: a multiple baseline study. Behav. Intervent. 20 (1), 37–49. 10.1002/bin.168

[B42] Cherney L. R. Erickson R. K. Small S. L. (2010). Epidural cortical stimulation as adjunctive treatment for non-fluent aphasia: Preliminary findings. J. Neurol. Neurosurg. Psych. 81, 1014–1021. 10.1136/jnnp.2009.184036 20667854

[B43] Ciallella J. R. Yan H. Q. Ma X. Wolfson B. M. Marion D. W. DeKosky S. T. (1998). Chronic effects of traumatic brain injury on hippocampal vesicular acetylcholine transporter and M2 muscarinic receptor protein in rats. Exp. Neurol. 152, 11e19. 10.1006/exnr.1998.6831 9682008

[B44] Coelho C. A. (2007). Management of discourse deficits following traumatic brain injury: Progress, caveats, and needs. Semin. Speech Lang. 28, 122–135. 10.1055/s-2007-970570 17427051

[B45] Cohen L. Dehaene S. Naccache L. Lehéricy S. Dehaene-Lambertz G. Hénaff M. A. (2000). The visual word form area: spatial and temporal characterization of an initial stage of reading in normal subjects and posterior split-brain patients. Brain 123 (2), 291–307. 10.1093/brain/123.2.291 10648437

[B46] Cohen L. Martinaud O. Lemer C. Lehéricy S. Samson Y. Obadia M. (2003). Visual word recognition in the left and right hemispheres: anatomical and functional correlates of peripheral alexias. Cerebr. Cortex. 13 (12), 1313–1333. 10.1093/cercor/bhg079 14615297

[B47] Conde-Guzón P. A. Conde-Guzón M. Bartolomé-Albistegui M. Quirós P. (2009). Perfiles neuropsicológicos asociados a los problemas del lenguaje oral infantil. Rev. Neurol. 48, 32–38. 10.33588/rn.4801.2008164 19145564

[B48] Crawford J. R. Garthwaite P. H. Porter S. (2010). Point and interval estimates of effect sizes for the case-controls design in neuropsychology: Rationale, methods, implementations, and proposed reporting standards. Cogn. Neuropsychol. 27 (3), 245–260. 10.1080/02643294.2010.513967 20936548

[B49] Cubo E. Fernández-Jaén A. Moreno C. Anaya B. González M. Kompoliti K. (2008). Donepezil use in children and adolescents with tics and attention-deficit/hyperactivity disorder: an 18-week, single-center, dose-escalating, prospective, open-label study. Clin. Ther. 30 (1), 182–189. 10.1016/j.clinthera.2008.01.010 18343255

[B50] De la Tremblaye P. B. Cheng J. P. Bondi C. O. Kline A. E. (2019). Environmental enrichment, alone or in combination with various pharmacotherapies, confers marked benefits after traumatic brain injury. Neuropharmacology 145 (Pt A), 13–24. 10.1016/j.neuropharm.2018.02.032 29499273

[B51] De Renzi E. Vignolo L. A. (1962). The token test: A sensitive test to detect receptive disturbances in aphasics. Brain 85, 665–678. 10.1093/brain/85.4.665 14026018

[B52] Dixon C. E. Bao J. Long D. A. Hayes R. L. (1996). Reduced evoked release of acetylcholine in the rodent hippocampus following traumatic brain injury. Pharmacol. Biochem. Behav. 53, 679e686. 10.1016/0091-3057(95)02069-1 8866972

[B53] Dixon C. E. Ma X. Marion D. W. (1997). Reduced evoked release of acetylcholine in the rodent neocortex following traumatic brain injury. Brain Res. 749, 127e130. 10.1016/S0006-8993(96)01310-8 9070636

[B54] Doyle R. L. Frazier J. Spencer T. J. Geller D. Biederman J. Wilens T. (2006). Donepezil in the treatment of ADHD-like symptoms in youths with pervasive developmental disorder: a case series. J. Atten. Disord. 2006 9 (3), 543–549. 10.1177/1087054705284091 16481671

[B55] Duff M. C. Stuck S. (2015). Paediatric concussion: Knowledge and practices of school speech-language pathologists. Brain Inj. 29 (1), 64–77. 10.3109/02699052.2014.965747 25313854

[B56] Dunn L. M. Dunn L. M. Arribas D. (2006). PPVT-III PEABODY. Test de vocabulario en imágenes Peabody (Madrid, España: TEA).

[B57] Ewing-Cobbs L. Barnes M. (2002). Linguistic outcomes following traumatic brain injury in children. Semin. Pediatr. Neurol. 9, 209–217. 10.1053/spen.2002.35502 12350042

[B58] Forkel S. J. Rogalski E. Sancho N. D. D’Anna L. Laguna P. L. Sridhar J. (2020). Anatomical evidence of an indirect pathway for word repetition. Neurology 94 (6), e594–e606. 10.1212/WNL.0000000000008746 31996450 PMC7136066

[B59] Foulon C. Cerliani L. Kinkingnehun S. Levy R. Rosso C. Urbanski M. (2018). Advanced lesion symptom mapping analyses and implementation as BCB toolkit. Gig. Sci. 7 (3), giy004. 10.1093/gigascience/giy004 PMC5863218 29432527

[B60] François C. Ripollés P. Bosch L. Garcia-Alix A. Muchart J. Sierpowska J. (2016). Language learning and brain reorganization in a 3.5-year-old child with left perinatal stroke revealed using structural and functional connectivity. Cortex 77, 95–118. 10.1016/j.cortex.2016.01.010 26922507

[B61] François C. Ripollés P. Ferreri L. Muchart J. Sierpowska J. Fons C. (2019). Right structural and functional reorganization in 4-year-old children with perinatal arterial ischemic stroke predict language production. eNeuro 6 (4). 10.1523/ENEURO.0447-18.2019 PMC6749144 31383726

[B62] Fridriksson J. Holland A. L. Beeson P. Morrow L. (2005). Spaced retrieval treatment of anomia. Aphasiology 19 (2), 99–109. 10.1080/02687030444000660 16823467 PMC1486764

[B63] Fridriksson J. Rorden C. Elm J. Sen S. George M. S. Bonilha L. (2018). Transcranial Direct Current Stimulation vs Sham Stimulation to Treat Aphasia After Stroke: A Randomized Clinical Trial. JAMA Neurol. 75 (12), 1470–1476. 10.1001/jamaneurol.2018 30128538 PMC6583191

[B64] Gardner R. A. Cubero N. S. (1981). Digits forward and digits backward as two separate tests: normative data on 1567 school children. J. Clin. Child Adolesc. Psychol. 10 (2), 131–135.

[B65] Gielow M. R. Zaborszky L. (2017). The Input-Output Relationship of the Cholinergic Basal Forebrain. Cell. Rep. 18 (7), 1817–1830. 10.1016/j.celrep.2017.01.060 28199851 PMC5725195

[B66] Gleichgerrcht E. Kocher M. Nesland T. Rorden C. Fridriksson J. Bonilha L. (2015). Preservation of structural brain network hubs is associated with less severe post-stroke aphasia. Restor. Neurol. Neurosci. 34 (1), 19–28. 10.3233/RNN-150511 26599472

[B67] Green-Heredia C. Sage K. Lambon Ralph M. Berthier M. L. (2009). Relearning verbal labels in a case of semantic dementia. Aphasiology 23, 192–209. 10.1080/02687030801942999

[B68] Halperin J. M. Healey J. M. Zeitchik E. Ludman W. L. Weinstein L. (1989). Developmental aspects of linguistic and mnestic abilities in normal children. J. Clin. Exp. Neuropsychol. 11 (4), 518–528. 10.1080/01688638908400910 2760184

[B69] Handen B. L. Johnson C. R. McAuliffe-Bellin S. Murray P. J. Hardan A. Y. (2011). Safety and efficacy of donepezil in children and adolescents with autism: neuropsychological measures. J. Child Adolesc. Psychopharmacol. 21 (1), 43–50. 10.1089/cap.2010.0024 21309696 PMC3037196

[B70] Hardan A. Y. Handen B. L. (2002). A retrospective open trial of adjunctive donepezil in children and adolescents with autistic disorder. J. Child Adolesc. Psychopharmacol. 12 (3), 237–241. 10.1089/104454602760386923 12427297

[B71] Hazell P. (2007). Drug therapy for attention-deficit/hyperactivity disorder-like symptoms in autistic disorder. J. Paediatr. Child Health 43 (1-2), 19–24. 10.1111/j.1440-1754.2007.00995.x 17207050

[B72] Heller J. H. Spiridigliozzi G. A. Doraiswamy P. M. Sullivan J. A. Crissman B. G. Kishnani P. S. (2004). Donepezil effects on language in children with Down syndrome: results of the first 22-week pilot clinical trial. Am. J. Med. Genet. A. 130A (3), 325–326. 10.1002/ajmg.a.30184 15378553 PMC2665884

[B73] Herbet G. Moritz-Gasser S. Lemaitre A. L. Almairac F. Duffau H. (2019). Functional compensation of the left inferior longitudinal fasciculus for picture naming. Cogn. Neuropsychol. 36(3–4), 140–157. 10.1080/02643294.2018.1477749 29879863

[B74] Hickok G. Poeppel D. (2000). Towards a functional neuroanatomy of speech perception. Trends. Cogn. Sci. 4 (4), 131–138. 10.1016/S1364-6613(00)01463-7 10740277

[B75] Hickok G. Poeppel D. (2007). The cortical organization of speech processing. Nat. Rev. Neurosci. 8 (5), 393. 10.1038/nrn2113 17431404

[B76] Hutsler J. J. Gazzaniga M. S. (1996). Acetylcholinesterase staining in human auditory and language cortices: regional variation of structural features. Cereb. Cortex. 6 (2), 260–270. 10.1093/cercor/6.2.260 8670655

[B77] Ivanova M. V. Isaev D. Y. Dragoy O. V. Akinina Y. S. Petrushevskiy A. G. Fedina O. N. (2016). Diffusion-tensor imaging of major white matter tracts and their role in language processing in aphasia. Cortex 2016, 85:165–85:181. 10.1016/j.cortex.2016.04.019 27289586

[B78] Jennett B. (1996). Epidemiology of head injury. J. Neurol. Neurosurg. Psych. 60, 362–369. 10.1136/jnnp.60.4.362 PMC1073884 8774396

[B79] Johnson C. J. Beitchman J. H. Brownlie E. B. (2010). Twenty-year follow-up of children with and without speech-language impairments: Family, educational, occupational, and quality of life outcomes. Am. J. Speech Lang. Pathol. 19 (1), 51–65. 10.1044/1058-0360(2009/08-0083) 19644128

[B80] Kaplan E. Goodglass H. Weintraub S. (1983). Boston Naming Test (Philadelphia: Lea and Febiger).

[B81] Kay J. Lesser R. Coltheart M. (1992). PALPA: Psycholinguistic Assessments of Language Processing in Aphasia Vol. Volume 2 (UK: Erlbaum).

[B82] Kertesz A. (1982). Western Aphasia Battery (New York: Grune and Stratton).

[B83] Kertesz A. (2007). Western Aphasia Battery: Revised (Pearson).

[B84] Kishnani P. S. Sullivan J. A. Spiridigliozzi G. A. Hellere J. H. Crissman B. G. (2004). Donepezil use in down syndrome. Arch. Neurol. 61 (4), 605–606. 10.1001/archneur.61.4.605-b 15096417

[B85] Kishnani P. S. Heller J. H. Spiridigliozzi G. A. Lott I. Escobar L. Richardson S. (2010). Donepezil for treatment of cognitive dysfunction in children with Down syndrome aged 10-17. Am. J. Med. Genet. A. 152A (12), 3028–3035. 10.1002/ajmg.a.33730 21108390

[B86] Kleim J. A. Jones T. A. (2008). Principles of experience-dependant neural plasticity: Implications for rehabilitation after brain damage. J. Speech Lang. Hear. Res. 51, S225–S239. 10.1044/1092-4388(2008/018) 18230848

[B87] Kurland J. Baldwin K. Tauer C. (2010). Treatment-induced neuroplasticity following intensive naming therapy in a case of chronic Wernicke’s aphasia. Aphasiology 24 (6-8), 737–751. 10.1080/02687030903524711

[B88] Laatsch L. Harrington D. Hotz G. Marcantuono J. Mozzoni M. P. Walsh V. (2007). An evidence-based review of cognitive and behavioral rehabilitation treatment studies in children with acquired brain injury. J. Head Trauma. Rehabil. 22 (4), 248–256. 10.1097/01.HTR.0000281841.92720.0a 17667068

[B89] Laine M. Martin N. (2006). Brain Damage, Behaviour, and Cognition Series. Anomia. Theoretical and Clinical Aspects (UK: Psychology Press).

[B90] Lassaletta A. Bouffet E. Mabbott D. Kulkarni A. V. (2015). Functional and neuropsychological late outcomes in posterior fossa tumors in children. Childs Nerv. Syst. 31 (10), 1877–1890. 10.1007/s00381-015-2829-9 26351237

[B91] Levin H. S. (2003). Neuroplasticity following non-penetrating traumatic brain injury. Brain Inj. 17, 665–674. 10.1080/0269905031000107151 12850951

[B92] López-Barroso D. de Diego-Balaguer R. Cunillera T. Camara E. Münte T. F. Rodriguez-Fornells A. (2011). Language learning under working memory constraints correlates with microstructural differences in the ventral language pathway. Cereb. Cortex. 21 (12), 2742–2750. 10.1093/cercor/bhr064 21527790

[B93] López-Barroso D. Catani M. Ripollés P. Dell’Acqua F. Rodríguez-Fornells A. Diego-Balaguer R. (2013). Arcuate fasciculus mediates word learning. PNAS 110 (32), 13168–13173. 10.1073/pnas.1301696110 23884655 PMC3740909

[B94] López-Barroso D. Thiebaut de Schotten M. T. Morais J. Kolinsky R. Braga L. W. Guerreiro- Tauil A. (2020). Impact of literacy on the functional connectivity of vision and language related networks. NeuroImage 213, 116722. 10.1016/j.neuroimage.2020.116722 32156625

[B95] Markello R. D. Spreng R. N. Luh W. M. Anderson A. K. De Rosa E. (2018). Segregation of the human basal forebrain using resting state functional MRI. Neuroimage 173, 287–297. 10.1016/j.neuroimage.2018.02.042 29496614

[B96] Martin N. Ayala J. (2004). Measurements of auditory-verbal STM span in aphasia: effects of item, task, and lexical impairment. Brain Lang. 89 (3), 464–483. 10.1016/j.bandl.2003.12.004 15120538

[B97] McKinnon E. T. Fridriksson J. Basilakos A. Hickok G. Hillis A. E. Spampinato M. V. (2018). Types of naming errors in chronic post-stroke aphasia are dissociated by dual stream axonal loss. Sci. Rep. 8 (1), 14352. 10.1038/s41598-018-32457-4 30254222 PMC6156587

[B98] Mena-Segovia J. Bolam J. P. (2017). Rethinking the Pedunculopontine Nucleus: From Cellular Organization to Function. Neuron 94 (1), 7–18. 10.1016/j.neuron.2017.02.027 28384477

[B99] Mesulam M. M. (2004). The cholinergic innervation of the human cerebral cortex. Prog. Brain Res. 145, 67–78. 10.1016/S0079-6123(03)45004-8 14650907

[B100] Moreno-Martínez F. J. Rodríguez-Rojo I. C. (2015). The Nombela 2.0 semantic battery: an updated Spanish instrument for the study of semantic processing. Neurocase 21 (6), 773–785. 10.1080/13554794.2015.1006644 25645383

[B101] Narbona J. Crespo-Eguilaz N. (2012). Brain plasticity for language in children and adolescents. Rev. Neurol. 54 (Suppl 1), S127–S130. 10.33588/rn.54S01.2012042 22374764

[B102] Noonan K. A. Jefferies E. Visser M. Lambon Ralph M. A. (2013). Going beyond inferior prefrontal involvement in semantic control: evidence for the additional contribution of dorsal angular gyrus and posterior middle temporal cortex. J. Cogn. Neurosci. 25 (11), 1824–1850. 10.1162/jocn_a_00442 23859646

[B103] Northam G. B. Adler S. Eschmann K. C. Chong W. K. Cowan F. M. Baldeweg T. (2018). Developmental conduction aphasia after neonatal stroke. Ann. Neurol. 83 (4), 664–675. 10.1002/ana.25218 29572915 PMC6681109

[B104] Nugiel T. Alm K. H. Olson I. R. (2016). Individual differences in white matter microstructure predict semantic control. Cogn. Affect. Behav. Neurosci. 16 (6), 1003–1016. 10.3758/s13415-016-0448-x 27444936 PMC5154852

[B105] Olabarrieta-Landa L. Rivera D. Rodríguez-Lorenzana A. Pohlenz Amador S. García-Guerrero C. E. Padilla-López A. (2017). Shortened Version of the Token Test: Normative data for Spanish-speaking pediatric population. NeuroRehabilitation 41 (3), 649–659. 10.3233/NRE-172244 28946594

[B106] Oldfield R. C. (1971). The assessment and analysis of handedness: The Edinburgh inventory. Neuropsychologia 9 (1), 97–113. 10.1016/0028-3932(71)90067-4 5146491

[B107] Osterrieth P. A. (1944). The test of copying a complex figure: A contribution to the study of perception and memory. Arch. Psychol. 30, 286–356.

[B108] Pityaratstian N. (2005). Advances in alternative pharmacotherapy of ADHD. J. Med. Assoc. Thai. 88 (Suppl 4), S357–S362. 16623055

[B109] Popper C. W. (2000). Pharmacologic alternatives to psychostimulants for the treatment of attention-deficit/hyperactivity disorder. Child Adolesc. Psychiatr. Clin. N. Am. 9 (3), 605–46, viii. 10.1016/S1056-4993(18)30109-3 10944659

[B110] Portellano J. A. Martínez-Arias R. Zumárraga L. (2009). Manual ENFEN, Evaluación Neuropsicológica de las Funciones Ejecutivas (Madrid.: TEA Ediciones).

[B111] Pulvermüller F. Berthier M. L. (2008). Aphasia therapy on a neuroscience basis. Aphasiology 22 (6), 563–599. 10.1080/02687030701612213 18923644 PMC2557073

[B112] Pulvermuller F. Neininger B. Elbert T. Mohr B. Rockstroh B. Koebbel P. (2001). Constraint-induced therapy of chronic aphasia after stroke. Stroke 32, 1621–1626. 10.1161/01.STR.32.7.1621 11441210

[B113] Rauschecker A. M. Deutsch G. K. Ben-Shachar M. Schwartzman A. Perry L. M. Dougherty R. F. (2009). Reading impairment in a patient with missing arcuate fasciculus. Neuropsychologia 47 (1), 180–194. 10.1016/j.neuropsychologia.2008.08.011 18775735 PMC2671152

[B114] Rey G. J. Feldman E. Rivas-Vazquez R. Levin B. E. Benton A. (1999). Neuropsychological test development and normative data on Hispanics. Arch. Clin. Neuropsych. 14 (7), 593–601. 14590573

[B115] Reynolds C. R. Bigler E. D. (1996). Factor Structure, Factor Indexes, and Other Useful Statistics for Interpretation of the Test of Memory and Learning (TOMAL). Arch. Clin. Neuropsychol. 11 (1), 29–43. 10.1093/arclin/11.1.29

[B116] Ripollés P. Biel D. Peñaloza C. Kaufmann J. Marco-Pallarés J. Noesselt T. (2017). Strength of temporal white matter pathways predicts semantic learning. J. Neurosci. 37 (46), 11101–11113. 10.1523/JNEUROSCI.1720-17.2017 29025925 PMC6596806

[B117] Rivera D. Morlett-Paredes A. Peñalver Guia A. I. Irías Escher M. J. Soto-Añari M. Aguayo Arelis A. (2017). Stroop color-word interference test: Normative data for Spanish-speaking pediatric population. NeuroRehabilitation 41 (3), 605–616. 10.3233/NRE-172246 28946595

[B118] Rojkova K. Volle E. Urbanski M. Humbert F. Dell’Acqua F. Thiebaut de Schotten M. (2016). Atlasing the frontal lobe connections and their variability due to age and education: a spherical deconvolution tractography study. Brain. Struct. Funct. 221 (3), 1751–1766. 10.1007/s00429-015-1001-3 25682261

[B119] Rokem A. Silver M. A. (2010). Cholinergic enhancement augments magnitude and specificity of visual perceptual learning in healthy humans. Curr. Biol. 20 (19), 1723–1728. 10.1016/j.cub.2010.08.027 20850321 PMC2953574

[B120] Rorden C. Brett M. (2000). Stereotaxic display of brain lesions. Behav. Neurol. 12, 191–200. 10.1155/2000/421719 11568431

[B121] Rothenberger A. (1986). Aphasia in children. Fortschr. Neurol. Psychiatr. 54 (3), 92–98. 10.1055/s-2007-1001855 2420691

[B122] Ryan N. P. Genc S. Beauchamp M. H. Yeates K. O. Hearps S. Catroppa C. (2018). White Matter Microstructure Predicts Longitudinal Social Cognitive Outcomes After Paediatric Traumatic Brain Injury: A Diffusion Tensor Imaging Study. Psychol. Med. 48 (4), 679–691. 10.1017/S0033291717002057 28780927

[B123] Sahu J. K. Gulati S. Sapra S. Arya R. Chauhan S. Chowdhury M. R. (2013). Effectiveness and safety of donepezil in boys with fragile x syndrome: a double-blind, randomized, controlled pilot study. J. Child. Neurol. 28 (5), 570–575. 10.1177/0883073812449381 22752489

[B124] Saur D. Kreher B. W. Schnell S. Kümmerer D. Kellmeyer P. Vry M.-S. (2008). Ventral and dorsal pathways for language. PNAS 105 (46), 18035–18040. 10.1073/pnas.0805234105 19004769 PMC2584675

[B125] Sedó M. A. (2004). The “Five Digit Test”: a color-free, non-reading alternative to the Stroop. Int. Neuropsychol. Soc. Liaison Committee Newslett. 13, 6–7.

[B126] Sedó M. A. (2007). FDT: test de los cinco dígitos. (Madrid: Tea Ediciones).

[B127] Sergui-Gomez M. MacKenzie E. J. (2003). Measuring the public health impact of injuries. Epidemiol. Rev. 25, 3–19. 10.1093/epirev/mxg007 12923986

[B128] Shaw K. E. Bondi C. O. Light S. H. Massimino L. A. McAloon R. L. Monaco C. M. (2013). Donepezil is ineffective in promoting motor and cognitive benefits after controlled cortical impact injury in male rats. J. Neurotrauma. 30, 557e564. 10.1089/neu.2012.2782 23227953 PMC3636588

[B129] Sierpowska J. Gabarrós A. Fernández-Coello A. Camins À. Castañer S. Juncadella M. (2019). White-matter pathways and semantic processing: intrasurgical and lesion-symptom mapping evidence. NeuroImage: Clin. 22, 101704. 10.1016/j.nicl.2019.101704 30743137 PMC6370559

[B130] Simić G. Mrzljak L. Fucić A. Winblad B. Lovrić H. Kostović I. (1999). Nucleus subputaminalis (Ayala): the still disregarded magnocellular component of the basal forebrain may be human specific and connected with the cortical speech area. Neuroscience 89 (1), 73–89. 10.1016/s0306-4522(98)00304-2 10051218

[B131] Slomine B. Locascio G. (2009). Cognitive rehabilitation for children with acquired brain injury. Dev. Disabil. Res. Rev. 15 (2), 133–143. 10.1002/ddrr.56 19489085

[B132] Snodgrass J. G. Vanderwart M. (1980). A standardized set of 260 pictures: Norms for name agreement, image agreement, familiarity, and visual complexity. J. Exp. Psych. 6 (2), 174–215. 10.1037/0278-7393.6.2.174 7373248

[B133] Spencer T. Biederman J. (2002). Non-stimulant treatment for Attention-Deficit/Hyperactivity Disorder. J. Atten. Disord. 6 (Suppl 1), S109–S119. 10.1177/070674370200601S13 12685525

[B134] Spiridigliozzi G. A. Heller J. H. Crissman B. G. Sullivan-Saarela J. A. Eells R. Dawson D. (2007). Preliminary study of the safety and efficacy of donepezil hydrochloride in children with Down syndrome: a clinical report series. Am. J. Med. Genet. A. 143A (13), 1408–1143. 10.1002/ajmg.a.31790 17542008

[B135] Srivastava R. K. Agarwal M. Pundhir A. (2011). Role of donepezil in autism: its conduciveness in psychopharmacotherapy. Case Rep. Psychiatry 2011:563204. 10.1155/2011/563204 22937405 PMC3420777

[B136] Stroop J. R. (1935). Studies of interference in serial verbal reactions. J. Exp. Psych. 18 (6), 643–662. 10.1037/h0054651

[B137] Suárez-González A. Green Heredia C. Savage S. A. Gil-Néciga E. García-Casares N. Franco-Macías E. (2015). Restoration of conceptual knowledge in a case of semantic dementia. Neurocase 21 (3), 309–321. 10.1080/13554794.2014.892624 24592963

[B138] Sullivan J. R. Riccio C. A. (2010). Language Functioning and Deficits Following Pediatric Traumatic Brain Injury. Appl. Neuropsych. 17 (2), 93–98. 10.1080/09084281003708852 20467948

[B139] Tamasaki A. Saito Y. Ueda R. Ohno K. Yokoyama K. Satake T. (2016). Effects of donepezil and serotonin reuptake inhibitor on acute regression during adolescence in Down syndrome. Brain Dev. 38 (1), 113–117. 10.1016/j.braindev.2015.06.006 26143664

[B140] Tavano A. Galbiati S. Recla M. Formica F. Giordano F. Genitori L. (2009). Language and cognition in a bilingual child after traumatic brain injury in infancy: Long-term plasticity and vulnerability. Brain Injury 23 (2), 167–171. 10.1080/02699050802657012 19191096

[B141] Teasdale G. Jennett B. (1976). Assessment and prognosis of coma after head injury. Acta Neurochir. (Wien). 34 (1-4), 45–55. 10.1007/BF01405862 961490

[B142] Thiebaut de Schotten M. Cohen L. Amemiya E. Braga L. W. Dehaene S. (2014). Learning to read improves the structure of the arcuate fasciculus. Cereb. Cortex. 24 (4), 989–995. 10.1093/cercor/bhs383 23236205

[B143] Thomas-Stonell N. Johnson P. Schuller R. Jutai J. (1994). Evaluation of a computer-based program for remediation of cognitive-communication skills. J. Head Trauma Rehab. 9 (4), 25–37. 10.1097/00001199-199412000-00005

[B144] Thornton S. L. Pchelnikova J. L. Cantrell F. L. (2016). Characteristics of Pediatric Exposures to Antidementia Drugs Reported to a Poison Control System. J. Pediatr. 172, 147–150. 10.1016/j.jpeds.2016.01.056 26935787

[B145] Torres-Prioris M. J. López-Barroso D. Càmara E. Fittipaldi S. Sedeño L. Ibáñez A. (2020). Neurocognitive signatures of phonemic sequencing in expert backward speakers. Sci. Rep. 10, 10621. 10.1038/s41598-020-67551-z 32606382 PMC7326922

[B146] Turkstra L. S. Politis A. M. Forsyth R. (2015). Cognitive-communication disorders in children with traumatic brain injury. Dev. Med. Child. Neurol. 57, 217–222. 10.1111/dmcn.12600 25283953

[B147] Valle F. Cuetos F. (1995). EPLA: Evaluación del Procesamiento Lingüístico en la Afasia (Hove, UK: Lawrece Erlbaum Associates).

[B148] Van Hout A. Evrard P. H. Lyon G. (1985). On the positive semiology of acquired aphasia in children. Dev. Med. Child. Neurol. 27, 231–241. 10.1111/j.1469-8749.1985.tb03774.x 2581836

[B149] Van Hout A. (1997). Acquired Aphasia in Children. Semin. Pediatr. Neurol. 4 (2), 102–108. 10.1016/S1071-9091(97)80026-5 9195667

[B150] Van Hout A. (2003). “Acquired aphasia in childhood,” in Handbook of Neuropsychology, (2nd ed. Eds. Segalowitz S. J. Rapin I. (Amsterdam: Elsevier Science), 631–658.

[B151] Vik A. Kvistad K. A. Skandsen T. Ingebrigtsen T. (2006). Diffuse axonal injury in traumatic brain injury. Tidsskrift. Norske. Laege-foren. 22, 2940–2944. 17117192

[B152] Walker W. Seel R. Gibellato M. Lew H. Cornis-Pop M. Jena T. (2004). The effects of Donepezil on traumatic brain injury acute rehabilitation outcomes. Brain. Injury. 18 (8), 739–750 . 10.1080/02699050310001646224 15204315

[B153] Warden D. L. Gordon B. McAllister T. W. Silver J. M. Barth J. T. Bruns J. (2006). Guidelines for the pharmacologic treatment of neurobehavioral sequelae of traumatic brain injury. J. Neurotrauma. 23, 1468e1501. 10.1089/neu.2006.23.1468 17020483

[B154] Wechsler D. (2014). Wechsler intelligence scale for children-fifth edition (Bloomington, MN: Pearson).

[B155] Whiting E. Chenery H. J. Chalk J. Copland D. A. (2007). Dexamphetamine boosts naming treatment effects in chronic aphasia. J. Int. Neuropsychol. Soc 13 (6), 972–979. 10.1017/S1355617707071317 17942015

[B156] Wilens T. E. Biederman J. Wong J. Spencer T. J. Prince J. B. (2000). Adjunctive Donepezil in Attention Deficit Hyperactivity. J. Child Adolesc. Psychopharmacol. 10 (3), 217–222. 10.1089/10445460050167322 11052411

[B157] Wiseman-Hakes C. Stewart M. L. Wasserman R. Schuller R. (1998). Peer group training of pragmatic skills in adolescents with acquired brain injury. J. Head Trauma Rehabil. 13 (6), 23–36. 10.1097/00001199-199812000-00005 9885316

[B158] Woods M. T. Teuber H. L. (1978). Changing patterns of childhood aphasia. Ann. Neurol. 3, 273–280. 10.1002/ana.410030315 666267

[B159] Yeatman J. D. Feldman H. M. (2013). Neural plasticity after pre-linguistic injury to the arcuate and superior longitudinal fasciculi. Cortex 49 (1), 301–311. 10.1016/j.cortex.2011.08.006 21937035 PMC3259257

[B160] Zhang X. Shu B. Zhang D. Huang L. Fu Q. Du G. (2018). The Efficacy and Safety of Pharmacological Treatments for Post-stroke Aphasia. CNS Neurol. Disord. Drug Targets. 17 (7), 509–521. 10.2174/1871527317666180706143051 29984673

