# Supplementary material for: Pharmacotherapy of Traumatic Childhood Aphasia: Beneficial Effects of Donepezil Alone and Combined With Intensive Naming Therapy
Source: Front Pharmacol. 2020 Jul 31;11:1144. doi: 10.3389/fphar.2020.01144 (PMC7411310; doi:10.3389/fphar.2020.01144)
Supplement: Supplementary file 1 [file DataSheet_1.docx]

**Supplementary Material**

1. **Supplementary Methods**

**NOMBELA 2.0 SEMANTIC BATTERY**

**Authors**: Moreno-Martínez, F.J & Rodríguez-Rojo, I.C

**Citation**: Moreno-Martínez, F. J., & Rodríguez-Rojo, I. C. (2015): The Nombela 2.0 semantic battery: an updated Spanish instrument for the study of semantic processing, *Neurocase: The Neural Basis of Cognition*, DOI: 10.1080/13554794.2015.1006644.

The Nombela 2.0 is a validated Spanish instrument for the assessment of semantic processing. Validation studies were only performed with adult population and, thereby, there is not normative data available for children. The battery includes both *living* beings (animals, body parts, flowers, fruits, insects, trees, and vegetables) and *non-living* things (buildings, clothing, furniture, kitchen utensils, musical instruments, tools, and vehicles). It consists of five tasks designed to explore category specificity by tapping semantic production and comprehension, using both visual and verbal input. The five tasks are: generation of verbal definition (14 stimuli), two naming tasks (picture naming and naming in response to verbal description; 98 stimuli), semantic fluency (14 semantic category) and word picture naming (42 stimuli). This neuropsychological tool was specifically constructed to present a low ceiling effect, even in healthy subjects.

**ENFEN - Neuropsychological Evaluation of Executive Functions in Children**

**Authors**: Portellano, J.A, Martínez-Arias, R., & Zumárraga, L.

**Citation**: Portellano, J.A., Martínez-Arias, R., Zumárraga, L. (2009). *Manual ENFEN, Evaluación Neuropsicológica de las Funciones Ejecutivas*. TEA Ediciones. Madrid.

The ENFEN is a battery that allows evaluating the global development of children during the schooling period, with a focus on executive functions. The ENFEN tests can be used to asses children between the ages of 6 and 12 as long as they do not have (1) severe cognitive disability and (2) sensory-motor disorders that prevent the execution of the test. Also, it is paramount for the children to have acquired basic reading abilities. The battery is composed of four tests that can be used in conjunction or independently: verbal fluency, trail, rings, and interference. All these tasks target different aspects of executive functions. For the purpose of the present study only the trail making, and interference subtests were used. The trail subtest is based on the well-known *Trail Making Test* and has two parts: the grey trail and the colour trail. In the grey trail the participant is asked to draw a line connecting numbers in descending order, going from 20 to 1, as rapidly in possible. The colour trail is composed of pink and yellow circled numbers and the participant is required to connect consecutive numbers (1 to 6) of different colours in an alternating sequence. The interference subtest consists on a set of 39 written colour names, each printed with an inconsistent colour ink (blue, red, yellow and green). Subjects are asked to name the colour of the ink in which the words are written rather than reading the words. This subtest is derived from the Stroop Test and allows assessing the ability to inhibit cognitive interference as well as selective attention and cognitive flexibility.

**Five-Digit Test**

**Author**: Sedó, M. A.

**Citation**: Sedo, M. A. (2004) The “Five Digit Test”: a colour-free, non-reading alternative to the Stroop. *International Neuropsychological Society Liaison Committee Newsletter*, 13, 6-7.

The five-digit test (FDT) is a very brief and simple tool for assessing cognitive processing speed, reorientation of attention and the ability to cope with interference. It is based on the well-known Stroop Test, but instead of using words and colours it works with figures and digits as stimuli. This allows for a greater variety of tests and can be used in people with a low cultural level who do not show high language skills or in illiterates. The test can be used with children as young as 7 years onwards. Correlations between the FDT and the Stroop were .71. Comparing neurotypical adults and stroke patients, the FDT classifies correctly 97.5% of the subjects. The FDT is the result of extensive research carried out by the author and other researchers in many different countries and has shown an excellent discrimination capacity, both in children and adults.

1. **Supplementary Results**

| **Table S1***.* Language Assessment. | | | | | | | |
| --- | --- | --- | --- | --- | --- | --- | --- |
|  | **Subject P** | | | | | **Reference value^a^** | |
|  | **LNE1**  t; p; Zcc | | **LNE2**  t; p; Zcc | **LNE3**  t; p; Zcc | **LNE4**  t; p; Zcc | **Mean** | **SD** |
| **Primary Outcome Measures** | | | | | | | |
| **Aphasia Quotient- Western Aphasia Battery-Revised (WAB-R AQ)** | **78.4***  -5.73; ≤ .001; -6.13 | | **92.6**  -.867; .210; -.927 | **95.8**  230; .413; .245 | **94.2**  -.319; .380; -.341 | **95.13** | **2.73** |
| Information Content**^§^**  Fluency**^§^**  Comprehension  Repetition  Naming | **8***  **8***  **8.5**  -.612; .282; -.654  **8**  -1.35; .113; -1.44  **6.7***  -3.30; .008; -3.53 | | **10**  **10**  **9.7**  .827; .220; .885  **9**  -.319; .380; -.341  **7.6***  -2.13; .038; -2.28 | **10**  **10**  **9.9**  1.07; .163; 1.14  **9.2**  -.113; .457; -.121  **8.8**  .572; .294; -.611 | **10**  **10**  **10**  1.07; .163; 1.14  **9**  -.319; .380; -.341  **8.1**  -1.48; .094; 1.58 | **10**  **10**  **9.01**  **9.31**  **9.24** | **0**  **0**  **.78**  **.91**  **.72** |
| **Secondary Outcome Measures** | | | | | | | |
| **Snodgrass and Vanderwart Object Pictorial Set (SVOPS)** | **138***  -8.47; ≤ .001; -9.05 | | **167***  -5.88; ≤ .001; -6.28 | **220**  -1.148; .147; -1.23 | **211***  -1.95; .049; -2.09 | **232.86** | **10.48** |
| **Nombela 2.0 Semantic Battery (NSB)**  Picture Naming  Semantic Fluency  Word-Picture Matching | **23***  -2.06; .042; -2.21  **56***  -2.13; .038; -2.28  **29 ***  -2.63; .019; -2.81 | | **39**  -.513; .313; -.549  **79**  -1.52; .089; -1.63  **32**  -1.60; .080; -1.71 | **51**  .651; .269; .696  **102**  -.916; .197; -.980  **35**  -.572; .294; -.612 | **53**  .845; .215; .904  **79**  -1.52; .089; -1.63  **34**  -.915; .197; -.978 | **44.29**  **136.71**  **36.67** | **9.64**  **35.43**  **2.73** |
| **Boston Naming Test (BNT)^1^** | **23***  -5.20; ≤.001; -5.32 | | **31***  -3.42; .001; -3.50 | **52**  1.24; .114; 1.27 | **40**  -1.42; .084; -1.46 | **46.41^b^** | **4.4^b^** |
| **Peabody: Picture Vocabulary Test III (PPVT-III)^2^** | **2** | | **42** | **63** | **39** | **standard scores^†^** | |
| **Token Test (Shortened version) (TT-sv)^3^** | **<5** | | **95** | **50** | **70** | **standard scores^†^** | |
| **Psycholinguistic Assessments of Language**  **Processing in Aphasia (PALPA)** | |  |  |  |  |  |  |
| Repetition: Nonwords (PALPA-8)  Repetition: Imageability x Frequency (PALPA-9)**^§^**  Repetition: Sentences (PALPA-12)  Reading: Visual Lexicon Decision (PALPA-25)  Reading: Grammatical Class (PALPA-32)**^§^**  Reading: Nonwords (PALPA-36)  Reading: Sentences (PALPA-37)**^§^**  Semantics: Spoken Word-Picture Matching (PALPA-47)  Semantics: Written Word-Picture Matching (PALPA-48)  Semantics: Spoken Word-Written Word Match (PALPA-52)  Semantics: Picture Naming (PALPA-53)  Semantics: Picture Naming x Frecuency (PALPA-54)  Spoken Sentence-Picture Matching (PALPA-55)  Written Sentence-Picture Matching (PALPA-56) | **16***  -8.79; ≤ .001; -9.39  **147***  **26***  -12.39; ≤ .001; -13.25  **138***  -4.15; .003; -4.43  **76***  **20***  -9.50; ≤ .001; -10.16  **33***  **36***  -2.90; .014; -3.10  **35***  -4.04; .003; -4.32  **33***  -2.17; .037; -2.32  **29***  -6.62; ≤ .001; -7.08  **53***  -5.11; .001; -5.47  **48***  -2.88; .014; -3.08  **46***  -3.37; .007; -3.60 | | **22**  -1.68;.072; -1.80  **160**  **23***  -16.46; ≤ .001; -17.59  **142***  -2.91; .013; -3.11  **75***  **19***  -11.96; ≤ .001; -12.79  **24***  **37**  -1.54; .086; -1.65  **40***  2.12; .039; 2.26  **32***  -2.74; .017; -2.93  **34***  -3.02; .012; -3.23  **55***  -3.37; .007; -3.60  **52**  -1.49; .094; -1.59  **48***  -2.63; .019; -2.81 | **24**  .687;.259; .734  -  **28***  -16.46; ≤ .001; -17.59  **138***  -4.15; ≤ .003; -4.43  **72***  **19***  -11.96; ≤ .001; -12.79  **32***  **39**  1.17; .144; 1.25  **38**  -.345;.371; -.368  **34**  -1.60; .081; -1.71  **36**  -1.58; .082; -1.69  **55***  -3.37; .007; -3.60  **53**  -1.14; .149; -1.22  **53**  -.788; .230; -.843 | **23**  -.497; .318; -.532  -  -  -  -  -  -  -  -  -  **38**  -.144; .445; -.154  -  **53**  -1.14; .149; -1.22  **52**  -1.16; .146; -1.24 | **23.42**  **160**  **35.14**  **151.43**  **80**  **23.86**  **36**  **38.14**  **38.28**  **36.80**  **38.20**  **58.85**  **56.28**  **55.14** | **.79**  **0**  **.69**  **3.03**  **0**  **.38**  **0**  **.69**  **.76**  **1.64**  **1.30**  **1.07**  **2.69**  **2.54** |
| **Controlled Oral Word Association Test (COWAT)** | **9**  -1.78; .063; -1.90 | | **23**  -.134; .449; -.143 | **15**  -1.07; .162; -1.15 | **17**  -.838; .217; -.896 | **24.14** | **7.97** |
| Asterisks (*) indicate significant differences at *p* < .05. Statistical comparisons were performed using one-tailed Crawford’s t-tests in all cases except for the tests marked with**^†^**. No statistical comparisons were performed for these tests since standardised scores provided a framework for comparing subject P´s performance against the normative data. Zcc is an effect size index proposed by Crawford, Garthwaite, and Porter (2010). ^a^Reference values represent the mean scores and standard deviations (SD) of the control group, except for ^b^, that represents the normative mean scores and SD provided by the test, and **^†^**^,^ that refer to normative data. ^†^The number indicated in each evaluation are the percentiles corresponding to the raw score obtained by subject P. **^1^**Normative data were obtained from: **^1^**Halperin et al. (1989), **^2^**Dunn, Dunn, & Arribas, (2006), and **^3^**Olabarrieta-Landa et al. (2017). ^§^Note that for these tests, SD of the control group mean equals zero, thereby there is no uncertainty about statistical significance and there is no need for performing a statistical test. | | | | | | | |
